# Rapid joule heating improves vitrification based cryopreservation

**DOI:** 10.1038/s41467-022-33546-9

**Published:** 2022-10-12

**Authors:** Li Zhan, Zonghu Han, Qi Shao, Michael L. Etheridge, Thomas Hays, John C. Bischof

**Affiliations:** 1 Department of Mechanical Engineering, University of Minnesota, Minneapolis, MN USA; 2 Department of Genetics, Cell Biology and Development, University of Minnesota, Minneapolis, MN USA; 3 Department of Biomedical Engineering, University of Minnesota, Minneapolis, MN USA; 4 Present Address: Center for Engineering in Medicine, Massachusetts General Hospital, Shriners Hospital for Children, Harvard Medical School, Boston, MA USA

**Keywords:** Biomedical engineering, Regenerative medicine, Mechanical engineering

## Abstract

Cryopreservation by vitrification has far-reaching implications. However, rewarming techniques that are rapid and scalable (both in throughput and biosystem size) for low concentrations of cryoprotective agent (CPA) for reduced toxicity are lacking, limiting the potential for translation. Here, we introduce a joule heating–based platform technology, whereby biosystems are rapidly rewarmed by contact with an electrical conductor that is fed a voltage pulse. We demonstrate successful cryopreservation of three model biosystems with thicknesses across three orders of magnitude, including adherent cells (~4 µm), *Drosophila melanogaster* embryos (~50 µm) and rat kidney slices (~1.2 mm) using low CPA concentrations (2–4 M). Using tunable voltage pulse widths from 10 µs to 100 ms, numerical simulation predicts that warming rates from 5 × 10^4^ to 6 × 10^8^ °C/min can be achieved. Altogether, our results present a general solution to the cryopreservation of a broad spectrum of cellular, organismal and tissue-based biosystems.

## Abstract (web summary)

Lower concentrations of toxic cryoprotective agents require rapid and scalable rewarming techniques. Here, the authors report on a joule heating–based platform where samples are rapidly rewarmed by contact with a voltage pulsed electrical conductor and demonstrate the preservation of *Drosophila* embryos and rat kidney slices.

## Introduction

Biological time can be effectively stopped when living biological systems are successfully cooled and stored at cryogenic temperatures (i.e., cryopreservation)^1,2^. Cryopreservation has brought massive societal and economic benefits by enabling the storage and transport of biological systems at multiple length scales ranging from cells to embryos and tissues. During cryopreservation, cryoprotective agents (CPAs) are loaded into the biosystems for protection against damage from ice crystallization. While routinely employed for cell cryopreservation, conventional convective slow cooling methods, which allow extracellular ice formation, provide poor viability for larger scale biosystems (i.e., *Drosophila melanogaster* embryos, coral larvae, tissues) due to chilling injury and/or intracellular ice formation^3–6^. Vitrification, an attractive ice-free alternative, turns liquid water to a glassy phase by cooling rapidly enough to avoid ice formation and chilling injury^7,8^. Indeed, vitrification has demonstrated superior outcomes in numerous biosystems compared with traditional slow cooling methods^9–13^. For vitrification, the sample cooling rate needs to exceed the critical cooling rate (CCR) of the loaded CPA to minimize ice formation. Likewise, to avoid devitrification, the rewarming rate needs to surpass the critical warming rate (CWR), which could be orders of magnitude higher than CCR^14–16^. In addition, as CPA concentration increases, the CCR and CWR decrease to more attainable values, but CPA toxicity increases. While vitrification has demonstrated proof of principle for a variety of biosystems, the potential translation for numerous important applications will only be realized once the technology can be scaled up in the throughput and/or size of biosystems. For example, high-throughput cryopreservation of *Drosophila* embryos could greatly improve the stock maintenance in labs and stock centers^17^. In addition, tissue slice cryopreservation provides a valuable tool for high-throughput drug screening^18,19^. However, with current approaches, it is challenging to achieve rapid, uniform, and scalable rewarming at low CPA concentrations for biosystems at different scales.

The traditional convective warming methods (i.e., rewarming in a water bath) suffers from the trade-off between sample geometry/scale and achievable warming rates^20–23^. Specifically, to achieve high warming rates, the sample size needs to remain small, limiting the throughput and translation. For larger sized biosystems, convective warming causes a large thermal gradient and stress between the center and edge. Furthermore, the achievable warming rates throughout a sample decrease with increasing size of the biosystem. To overcome these limitations of convective warming, numerous rapid warming approaches have been developed to improve the warming rate and uniformity. One common strategy, pioneered by Fahy and Mazur et al. introduces a controllable heat source either within (i.e., internal heating) or directly adjacent to (i.e., boundary heating) the biological system of interest. The achievable warming rate is positively correlated with the reported specific absorption rate (SAR, W/m^3^, Table 1). For internal heating, radiofrequency (RF) excited silica-coated iron oxide nanoparticles (sIONP) were included in a CPA solution to generate a warming rate > 300 °C/min^20,22,24,25^. In addition, microwave was used to directly rewarm tissues and CPA solution via dielectric coupling in the electromagnetic fields, achieving up to 150–300 °C/min warming rates^26–29^. In another approach, a focused halogen IR lamp is used to rewarm adherent cells via infrared radiation of water, with achievable warming rates of 2000–30,000 °C/min^29^. Furthermore, pulsed lasers coupled with gold nanorods (GNRs), or other laser absorbing materials, can successfully rewarm mouse oocytes, zebrafish embryos, coral larvae, and stem cells at rates ~10^7^ °C/min^3,23,30,31^. For boundary heating, tissues such as arteries with 1–2 mm thickness were placed in contact with metal forms and rewarmed in an alternating magnetic field (AMF), which inductively heated the metal, demonstrating a warming rate of up to ~2000 °C/min^21^.

**Table 1:** Summary of rewarming methods*

Warming method	Mechanism	SAR reported (W/m^3^)	Heating volume/area	Biomaterials tested & thickness	Warming rate (°C/min)	Ref.
RF nanowarming	RF heating of IONP	2.5 × 10^6^	1–80 mL	Porcine arteries (~0.7 mm) Porcine heart valve (~1.4 mm)	>300	^20,24,38^
Metal form	RF heating of Al foil	3.5−6.5 × 10^8^	1.3 cm × 2.8 cm	Porcine arteries (1-2 mm)	1.3−2 × 10^3^	^21^
Focused IR light	IR absorption of water	NA	200 µL	Adherent cells (~4 µm)	2 − 30 × 10^3^	^29^
Laser nanowarming	Laser plasmonic heating	4.4 × 10^11^	<10 µL droplet	Coral larvae (~100 µm) Cells in suspension (~15 µm)	7 × 10^4^–1.2 × 10^7^	^3,23,30^
Joule heating	Electrical current heating	3–600 × 10^11^	15 cm × 1 cm	Adherent cells (~4 µm) *Drosophila* embryo (~50 µm) Kidney slices (~1.2 mm)	5 × 10^4^–6.8 × 10^8^	This work

^*^Traditional convective warming using water bath is not listed here.

*RF* Radio frequency, *IONP* Iron oxide nanoparticle, *Al* Aluminum, *IR* Infrared radiation, *SAR* Specific absorption rate.

The thermal gradient inside biosystems is as important as the rapid warming rates and needs to be minimized to avoid thermal stress and cracking in the glassy phase. For boundary heating, the thermal gradient is governed by the size of biosystems and warming rate of heat source. Specifically, if the heat source is rewarmed too fast, large thermal gradients will form within the biosystem. For existing boundary heating approaches, a narrow range of warming rates of the heat sources was reported, limiting the potential to modulate the temperature uniformity. Additionally, depending on the heat generation mechanism, all of these rewarming approaches can only be applied to biosystems with a limited range of scales (Table 1). For instance, as a laser beam size is often on the order of millimeter, laser nanowarming is most suitable for small sized biosystems and allows relatively low CPA concentrations (i.e., < 4 M)^30^. We previously demonstrated that up to ~10 µL droplets can still be rapidly rewarmed using laser nanowarming^23^. In contrast, metal form and IONP nanowarming techniques can accommodate larger scale biosystems (i.e., tissues) using a scaled-up RF coil, albeit at slower warming rates (Table 1) and higher CPA concentration^20,21^.

Direct ohmic heating of a conductor can achieve ultrahigh warming rates under a voltage pulse^32,33^. In addition, the joule heating process can be precisely controlled by the voltage and pulse duration and convert almost 100% of the input electrical energy into thermal energy. Because of the direct conversion of electrons to heat, this technique yields the fastest possible rates of rewarming at the boundary of a resistive material. Importantly, scaling up joule heating is straightforward.

In this work, we report a format of rapid rewarming using joule heating to achieve rapid and scalable rewarming with low CPA concentrations for cryopreservation of biosystems at different scales. We demonstrate successful cryopreservation of three model biosystems with different thicknesses, including adherent cells (~4 µm), *Drosophila* embryos (~50 µm), and rat kidney slices (~1.2 mm), using low CPA concentrations (2–4 M). We perform scaling analysis and numerical modeling to rationally design the joule heating to achieve rapid rewarming, followed by experimental validation. Comparison with conventional convective warming methods demonstrate that joule heating greatly improved viabilities at lower CPA concentrations for all the model biosystems studied.

## Results

### Design of biosystem rewarming using joule heating

**Fig. 1:**
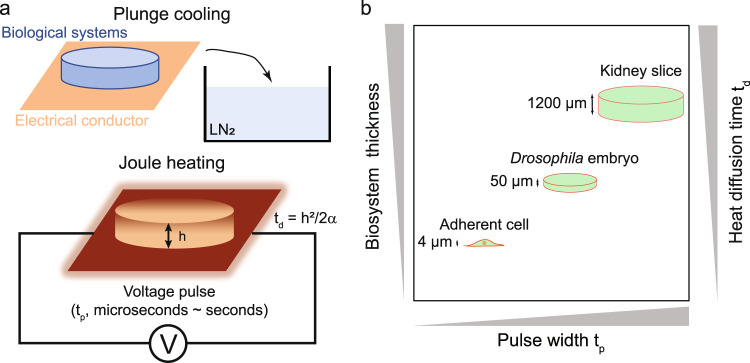
A rapid and scalable rewarming platform technique using joule heating. **a** Schematics of biological systems cryopreservation using plunge cooling and joule heating. The biological systems loaded with cryoprotective agent (CPA) were in contact with the electrical conductor. After removing the excess CPA solution, the biological systems and electrical conductor were plunged together into liquid nitrogen (LN_2_) for cooling. For rewarming, the electrical conductor was connected to a voltage pulse generator and generated heat via joule heating. The biomaterial was rewarmed via conduction. Drawings not to scale. **b** Three types of biosystems with different thicknesses were used as the model systems, including adherent cells (4 µm), *Drosophila* embryos (50 µm), and kidney slices (1.2 mm), as shown in the plot (drawings not to scale). The heat diffusion time (*t*_*d*_, right y-axis) across the biosystems is positively correlated with the biosystem thickness.

We sought to achieve rapid rewarming of biosystems in a simple manner using a boundary heat source (i.e., adjacent to the biosystems) by joule heating. The biosystems were in contact with an electrical conductor and rewarmed by heat conduction (Fig. 1a). The rates achievable within the biosystem were limited by the rate of heat diffusion, with slower warming rates experienced by the biosystems at locations further from the heat source. To investigate whether joule heating can serve as a platform technology to achieve rapid warming rates in a wide range to rewarm biosystems at different scales (i.e., thicknesses), we selected the adherent fibroblast cells (~ 4 µm), *Drosophila* embryos (~ 50 µm), and rat kidney slices (~ 1.2 mm) as the model systems (Fig. 1b). VS55 has been extensively used as the CPA for various biosystems^20–22^ and was used to cryopreserve adherent cells and kidney slices in this study. For *Drosophila* embryos, EG/sorbitol showed successful cryopreservation in our previous work and was therefore used here^17^. During cryopreservation, excess CPA solution was removed after CPA loading to reduce the thermal mass and improve the cooling and warming rates^17,34^. Rapid cooling was achieved by direct plunging into liquid nitrogen (Fig. 1a). The electrical conductor was then connected to a voltage pulse generator for rewarming. Using voltage pulse generators with a wide range of pulse widths (microseconds to seconds and longer) and voltage (several volts to thousands of volts and higher), adjustable warming rates across a wide range can be achieved. In general, higher rewarming rates of biosystems can be achieved by using shorter pulse widths.

### Characterization of joule heating in the RC discharge circuit

**Fig. 2:**
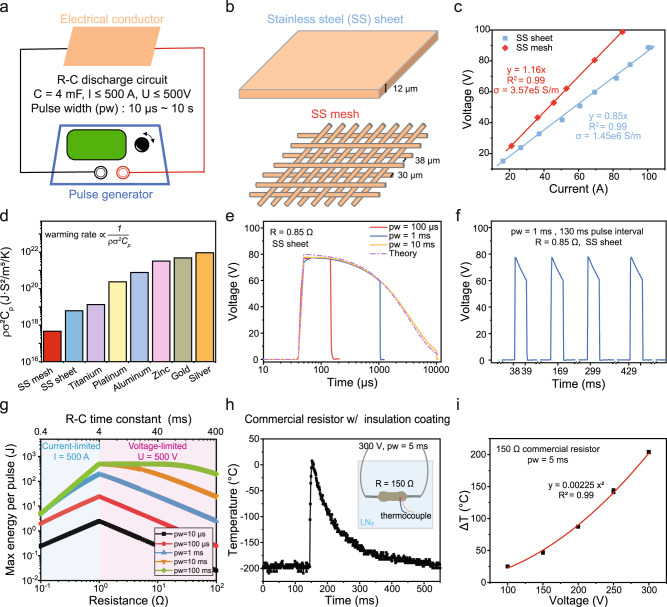
Characterization of joule heating using a pulse generator. **a** The R-C discharge circuit is used to generate joule heating by connecting the electrical conductor to a pulse generator. The voltage and pulse width can be adjusted. The capacitance (*C*) is 4000 µF. Ranges of the current (*I*), voltage (*V*), and pulse width (pw) for the low voltage mode (LV, ≤ 500 V) are listed in the figure. **b** Stainless steel (SS) sheet and mesh were used in this study. The thickness of SS sheet is 12 µm. The wire diameter and aperture size of the SS mesh are 30 and 38 µm, respectively. The SS mesh allows easy removal of excess CPA solution. **c** The electrical conductivity (*σ*) of the SS sheet and mesh can be calculated from the resistance by measuring the voltage and current. *n* = 6 independent experiments. **d** Comparison of various materials for rapid warming rate. The warming rate is inversely correlated to material density (*ρ*), electrical conductivity (*σ*), and heat capacity (*C*_*p*_). Details in Eq. 7 assuming same electrical current, material dimensions and pulse width. **e** Voltage profile when different voltage pulse widths were used. The exponential decay of measured voltage matched with the calculated voltage of a R-C discharge circuit. **f** Voltage profile when multiple voltage pulses were applied. **g** Maximum energy delivered per pulse depends on the resistance and pulse width. For *R* ≤ 1 Ω, the max current of 500 A is used for calculation. For *R* ≥ 1 Ω, the max voltage of 500 V is used for calculation. **h** Temperature profile of a thin film resistor (150 Ω) when subjected to a voltage pulse of 300 V, 5 ms. A thermocouple was attached to the electrically insulated surface of the resistor. The resistor was submerged in liquid nitrogen (LN_2_) during the measurement. Sampling resolution was 1 ms. **i** Temperature change (ΔT) as a function of applied voltage. The same setup shown in **h** was used. *n* = 6 independent experiments. For **c** and **i**, data presented as mean ± s.d.

To generate controllable rapid joule heating, we selected the resistor-capacitor (RC) discharge circuit using a voltage pulse generator (ECM® 830, BTX® Harvard Apparatus) that can produce pulse widths ranging from 10 µs to 10 s. The capacitor was charged to a preset voltage and energy was dissipated to the resistor (i.e., electrical conductor in Fig. 2a) via joule heating within the preset pulse width during the discharge phase. The voltage and pulse width could be adjusted. The low voltage (LV) mode of the pulse generator was used, with maximum voltage of 500 V and maximum current of 500 A. The capacitance was 4000 µF. To select the electrical conductors, we estimated the warming rate by assuming ohmic energy dissipation is converted to the thermal energy of the conductor within the voltage pulse (Eqs. 4–7). Under the same electrical current, material dimensions and pulse width, we found that warming rate is inversely correlated to three material properties including density (*ρ*), electrical conductivity (*σ*), and heat capacity (*C*_*p*_). As shown in Fig. 2d, among the commonly used metal materials, stainless steel (SS) provides the highest warming rate (i.e., lowest value of *ρσ*^2^ *C*_*p*_). In addition, the low cost and good biocompatibility of SS have been well documented^35,36^. We used a SS sheet (12 µm thickness) and mesh (wire diameter 30 µm, aperture size 38 µm) in this study, as shown in Fig. 2b. The SS sheet permitted cell attachment and was used for cryopreservation of adherent cells (i.e., human dermal fibroblast in this study). The SS mesh allowed easy removal of excess CPA solution to minimize the thermal mass for improved cooling and warming of the biosystems (i.e., *Drosophila* embryos and kidney slices in this study). Considering the woven format of the SS mesh, the effective electrical conductivity was obtained from voltage-current (*U-I*) measurements to facilitate numerical modeling of the joule heating process (Eqs. 2–11). Specifically, a section of SS mesh (12 cm length by 1 cm width) was subjected to different voltages, and the current was measured (Fig. 2c). The resistance (*R*) and electrical conductivity (*σ*) were obtained from the *U-I* correlation. The same measurement was performed on a SS sheet (15 cm length by 1 cm width). The measured electrical conductivity of the SS sheet was 1.45 × 10^6^ S/m, matching the values from the literature^37^. The SS mesh showed a lower effective electrical conductivity of 3.57 × 10^5^ S/m.

We next investigated the voltage profile and energy delivery ability of the pulse generator. The SS sheet (*R* = 0.85 Ω) was subjected to 80 V with different pulse widths (100 µs, 1 ms, and 10 ms). The RC time constant of the discharging capacitor was 3.4 ms (i.e., 0.85 Ω × 4 mF). The recorded voltage profile showed an exponential decay within the pulse width and good agreement with theoretical estimates (Fig. 2e). At the timepoint of 5* RC time constant, the voltage drops to 0.7% (i.e., e^−5^) of the peak value, suggesting that the feasible range of the pulse width is resistance dependent. Although one single voltage pulse was used in this study for cryopreservation, we showed that multiple pulses with a preset interval can be realized in high fidelity using this pulse generator (Fig. 2f). The decaying voltage implied a potentially decreasing warming rate during the pulse when the pulse width is larger than the RC time constant, which can be readily addressed by applying a square-wave voltage pulse in the future.

To understand the maximum energy each voltage pulse can deliver for different pulse widths, we identified the resistance of 1 Ω as the boundary between current-limited and voltage-limited regions. For example, given the pulse generator has a maximum voltage of 500 V and maximum current of 500 A, for a conductor with < 1 Ω resistance, the current limit of 500 A will be reached before the voltage limit of 500 V. According to Eqs. 2–3, the maximum energy per pulse was plotted with the resistance and pulse width as shown in Fig. 2g. For a given resistance, the max energy per pulse increases with pulse width and reaches the plateau once the pulse width exceeds 5 * RC time constant (upper x-axis in Fig. 2g). This is due to the completion of capacitive discharge before the pulse width ends.

In addition, we found that, compared to other resistance values, the resistance of 1 Ω produced the highest energy for different pulse widths when complete discharge of the capacitor was not achieved (i.e., pulse width < 5* RC time constant). When complete discharge occurred (i.e., the horizontal portion of the green line in Fig. 2g), the max energy per pulse remained the same, and the resistance varied from 1 Ω to approximately 10 Ω. These findings can guide the selection of resistance value for the cryopreservation of various biosystems using different pulse widths. For example, to achieve optimal energy delivery, for pulse widths < 10 ms, a resistance value around 1 Ω is preferred (orange line in Fig. 2g); for a pulse width of 100 ms, the optimal resistance value is more flexible (green line in Fig. 2g). Furthermore, the maximum energy delivered from the pulse generator per pulse was 500 J, as shown in Fig. 2g, which matched with the energy stored in a fully charged capacitor of 500 V (i.e., $\frac{1}{2}C{V}^{2}$).

To validate the joule heating performance, we measured the temperature change of the conductor after one voltage pulse. When the thermocouple was in contact with the SS sheet or mesh for direct temperature measurement, the applied voltage pulse interfered with the thermocouple output voltage signals which are ~50 µV/°C. Therefore, we measured the temperature of a commercial thin film resistor with an electrically insulated coating. Specifically, an ultrafine gauge thermocouple was attached to the surface of a 150 Ω thin film resistor which was connected to the pulse generator. The resistor and thermocouple were submerged in liquid nitrogen during the entire measurement. Figure 2h presented the measured temperature profile with 1 ms resolution when a 300 V, 5 ms voltage pulse was applied. We observed that the temperature of the resistor rapidly increased from −196 °C to 7.6 °C within 5 ms (warming rate of 2.4 × 10^6^ °C/min), and then decreased to −196 °C. These results demonstrated the physical feasibility of achieving rapid warming in a cryogenic object with joule heating using a pulse generator. Further, we showed that the temperature increase can be controlled with high consistency by adjusting the pulse voltage (Fig. 2i), which was found to be proportional to the square of the voltage, as suggested by the theoretical calculation (Eq. 2). Finally, as we will show, heating can also be accomplished using a sandwich system thereby increasing thickness and homogeneity in thicker systems.

### Joule heating for adherent cell cryopreservation

**Fig. 3:**
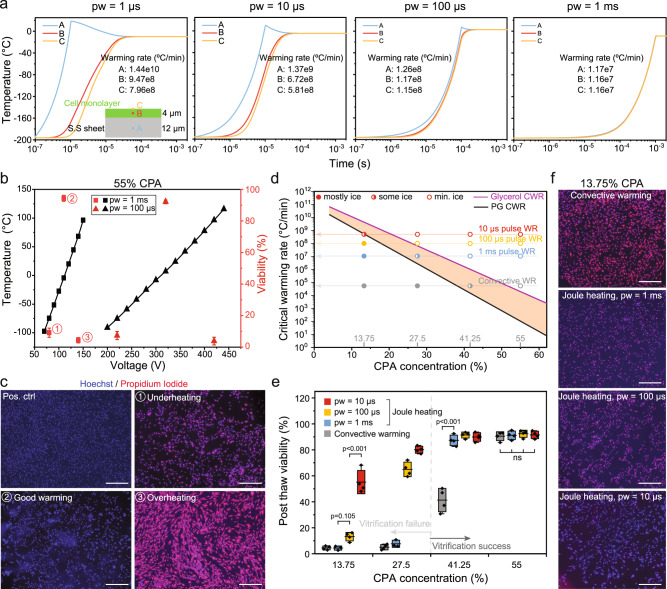
Adherent cells cryopreservation using joule heating. **a** Simulated temperature profile of stainless steel (SS) sheet and cell monolayer under pulse width (pw) ranging from 1 µs to 1 ms (shared y axis). The warming rates at the middle of SS sheet (point A), middle of cells (point B), and top of cells (point C) are displayed. **b** Simulated temperature (in black) and measured viability (in red) of human dermal fibroblasts (HDFs) for 1 ms and 100 µs pulse width with different voltages. VS55 (i.e., 55 wt%) was used as the cryoprotective agent (CPA). *n* = 4 independent experiments. Data presented as mean ± s.d. **c** Merged Hoechst/Propidium Iodide (PI) images of HDF cells. Conditions including underheating, good warming, overheating (for joule heating, as numbered in **b**), and positive control are shown. The pulse width is 1 ms. Scale bar is 500 µm. **d** Estimated critical warming rate (CWR) plotted as a function of CPA concentration. Propylene glycol (PG, good glass-forming tendency) and glycerol (poor glass-forming tendency) were used to estimate the range of CWR (orange area) of different CPA concentrations. In this study, 13.75%, 27.5%, 41.25%, and 55% CPA were tested. The measured convective warming rate (WR) were marked in the plot, along with the simulated WR of 1 ms, 100 µs, and 10 µs pulse joule heating. The experimentally tested conditions were labelled as dots at the intersections of CPA concentrations and warming rates. **e** Post-thaw viability of HDF cells using different CPA concentrations and warming methods. Vitrification failure (i.e., ice formation) was noted for 13.27% and 27% CPA groups. *n* = 4 independent experiment. Bounds and horizontal line of box represent standard deviation and mean, respectively; whiskers represent max and min. **f** For 13.75% CPA, merged Hoechst/PI images of HDF cells rewarmed by convective warming, 1 ms, 100 µs, and 10 µs pulse joule heating. Scale bar is 500 µm. One-way ANOVA and Tukey’s post hoc were used for statistical analysis. ns, *p* > 0.05.

After physical characterization of the joule heating approach, we explored application for adherent cells cryopreservation. Human dermal fibroblasts (HDFs) were seeded on a SS sheet (12 µm thickness,15 cm length, 1 cm width, *R* = 0.85 Ω) and incubated overnight for attachment. While cryopreservation of HDFs is routinely achieved through traditional slow-cooling methods, we chose HDFs as a well-characterized model system to study the cryopreservation response at the single-cell scale. In this study, we define biosystem heat diffusion length (*h*) as the distance within the biosystem across which heat needs to diffuse from the boundary heater (i.e., stainless steel sheet or mesh in this study) to rewarm the biosystem. Adherent cells are ultrathin (~4 µm) and therefore could achieve ultrarapid heat diffusion and warming rates. We first performed heat transfer simulations to estimate the warming rates for different pulse widths (1 µs, 10 µs, 100 µs, and 1 ms). The RC time constant was 3.4 ms, exceeding the tested pulse widths. In Fig. 3a, the simulated temperature profile and warming rate at the middle of the SS sheet (point A), middle of adherent cells (point B), and top of adherent cells (point C) are presented. For 10 µs pulse, the specific absorption rate (SAR) and warming rate can reach 5.9 × 10^13^ W/m^3^ and 6.7 × 10^8^ °C/min (for point B), respectively (Fig. 3a, Supplementary Fig. 1). The warming rate of the conventional convective warming method was measured to be 5.6 × 10^4^ °C/min (Supplementary Fig. 2). The heat diffusion time (*t*_*d*_ = *h*^2^/2*α*, where *h* is the biosystem heat diffusion length, *α* is thermal diffusivity) across the cell was estimated to be 8 µs. For a 1 µs pulse (i.e., < *t*_*d*_), although the SS sheet was rewarmed 10 times faster than a 10 µs pulse, the middle and top of cells (point B and C) were only rewarmed 1.4 times faster, due to conductive effects through the system as expected (Fig. 3a). In contrast, as pulse width increased beyond *t*_*d*_, the warming rate of the SS sheet more closely matched that of the cells (Fig. 1a).

To investigate the effect of warming rates on cell viability, we tested cryopreservation of adherent HDFs using 10 µs, 100 µs, and 1 ms pulse joule heating and conventional convective warming approaches. Different dilutions (25%, 50%, 75%, and 100%) of VS55 were used^38^. For example, 100% VS55 is 55 wt% CPA (i.e., 8.4 M) and 25% VS55 is 13.75 wt% CPA (i.e., 2.1 M). We loaded and unloaded the CPA in a stepwise manner on ice^11^. After CPA loading, excess CPA solution was removed by wicking methods prior to cooling. The cooling rate (i.e., repeatable plunge cooling procedure) was kept the same (1.9 × 10^4^ °C/min, Supplementary Fig. 2) for all conditions. To optimize the voltage for different pulse widths, the final temperatures of the cells after joule heating were modeled (Fig. 3b). A higher voltage was required for the shorter pulse width to achieve the same final temperature (Fig. 3b). For 100 µs and 1 ms pulse, we used VS55 to test the cell viability in the following conditions: (1) underheating, final temperature < −60 °C; (2) target warming temperature ~0 °C; and (3) overheating, final temperature > 60 °C. Hoechst/Propidium iodide (PI) membrane integrity dye was used as the viability indicator. As shown in Fig. 3b, c, high viability was obtained for the target warming condition, while the viability was very low for both underheating and overheating conditions.

Using the optimized voltages, the CPA concentration was also varied to investigate the impact on viability. In Fig. 3d, we plotted the estimated ranges of CWR for the CPA concentrations studied. Specifically, propylene glycol (PG, good glass-forming tendency) and glycerol (poor glass-forming tendency) were used as the boundaries for the estimated CWR regions (in orange). The CWR of PG and glycerol were extrapolated from measurements using the rapid laser calorimetry method^16^. In Fig. 3d, we marked the warming rates of different approaches and identified the intersections of different CPA concentrations with those rates, which represented various experimental conditions shown in Fig. 3e. Depending on whether the warming rate of the experimental condition is located above, within or below the CWR region, we predict minimal (i.e., success), moderate (i.e., marginal success) or notable (i.e., failure) ice formation associated with the warming processes, respectively. For instance, with 75% VS55, the warming rates for all joule heating conditions (10 µs, 100 µs, and 1 ms) were above the CWR region, indicating rewarming success. However, the warming rate for convective warming was within the CWR region, implying possible ice formation during rewarming. In addition, vitrification failure was noted for 25% VS55 and 50% VS55 CPAs during cooling.

The cell viability results in Fig. 3e illustrates the significance of rapid warming rates even under crystallized conditions. We found that the viability was improved with a faster warming rate when ice was expected during the cooling or warming phases. Even for 13.75% CPA, where ice crystallization is expected, 10 µs joule heating provided 55.2 ± 7.8% viability. In comparison, all the other warming conditions resulted in very low (< 5%) viability for the 13.75% CPA conditions as indicated in Fig. 3e, f. To the best of our knowledge, the estimated warming rate of 6.7 × 10^8^ °C/min for 10 µs joule heating represents the fastest warming rate achieved for cryopreservation rewarming (Table 1)^39,40^, allowing the rescue of adherent cells with intracellular ice during rapid rewarming. With an increased portion of ice formed during cooling (i.e., 25% VS55 vs. 50% VS55), the achieved fast warming rate may alleviate but not overwrite the ice injuries (Fig. 3e).

### Joule heating for *Drosophila* embryos cryopreservation

**Fig. 4:**
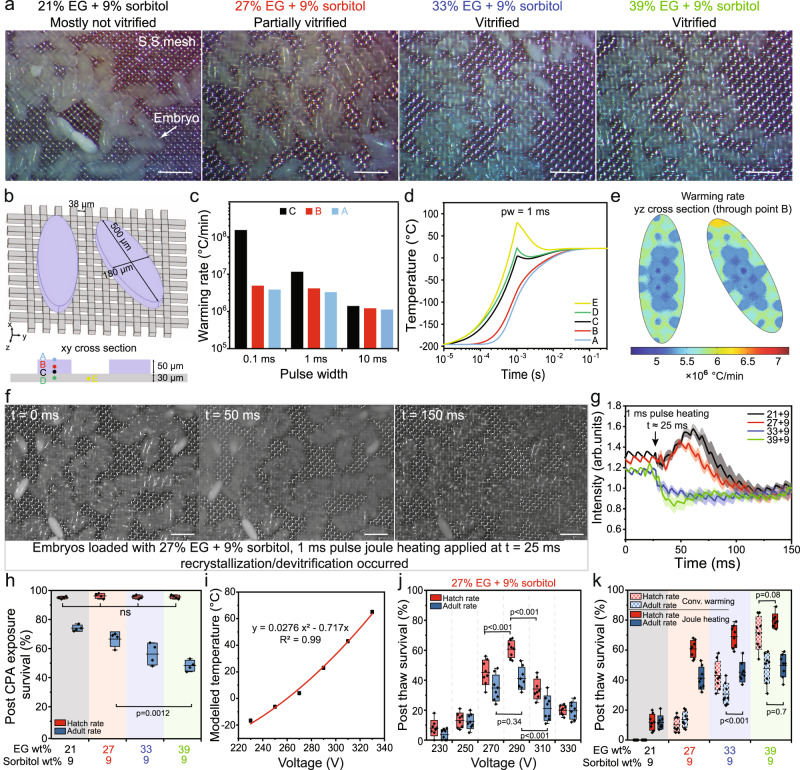
*Drosophila* embryos cryopreservation using joule heating. **a** Images of the *Drosophila* embryos on the stainless steel (SS) mesh after cooling. Embryos were loaded with different ethylene glycol (EG) concentrations. **b** Geometry and dimensions of the SS mesh and embryos used in the heat transfer modeling. Points A, B, and C represent the top, middle and bottom of the embryo, respectively. Point D and E represent the SS mesh in contact with the embryo and outside the embryo, respectively. **c** Temperature profile at different locations for 1 ms joule heating. **d** Warming rate distribution at the middle plane of embryos. **e** Warming rate of the embryo for different joule heating pulse widths. **f** Images of embryos on the SS mesh acquired by a high-speed camera at 3000 fps. The cryoprotective agent (CPA) was 27% EG + 9% sorbitol. The voltage was 290 V. **g** Normalized grayscale intensity of the embryos was plotted from the high-speed camera videos. Ice (i.e., white color) showed a high intensity value. Different CPA concentrations (labelled as EG + sorbitol concentration) were tested. *n* = 5. Data presented as mean ± s.d. **h** Survival of embryos after exposure to different CPA concentrations. Hatch and adult rates represent the survival from embryos to larvae and larvae to adults, respectively. *n* = 4. **i** Temperature of the embryos after 1 ms joule heating using different voltages. **j** Survival of embryos after 1 ms joule heating using different voltages. The CPA was 27% EG + 9% sorbitol. *n* = 8. **k** Comparison of embryo survival using convective warming and joule heating (290 V, 1 ms) for different CPA concentrations. *n* = 8. For **a** and **f**, scale bar is 500 µm. For **g**–**j**, *n* represent independent experiments. For **h**, **j**, and **k**, bounds and horizontal line of box represent standard deviation and mean respectively; whiskers represent max and min. For **c**–**e**, **i** modeling results were shown. Multivariate analysis of variance (MANOVA) and Tukey’s post hoc were used for statistical analysis. ns, *p* > 0.05.

Having validated the joule heating cryopreservation approach in adherent cells, we next examined its performance in cryopreserving *Drosophila* embryos, a more sophisticated and larger (i.e., 50 µm thickness after CPA loading) model system. We have previously reported successful recovery of adult flies from cryopreserved embryos with a cryomesh-based convective warming approach (2.2 × 10^5^ °C/min) using a nylon mesh and 39% EG + 9 % sorbitol as the CPA^17^. To explore the capability to achieve cryopreservation with lower CPA concentrations in this model system, we compare the post-thaw survival rates between our prior convective warming methods and joule heating using the SS mesh. A wildtype stock named WC1b (details in the Methods) was used. The woven SS mesh with 30 µm wire diameter and 38 µm aperture was used (Fig. 4a). We tested four CPA concentrations including 21%, 27%, 33%, and 39 wt% EG, supplemented with 9 wt% sorbitol. For CPA loading, embryos were equilibrated in 13% EG and then dehydrated with the higher CPA concentration mentioned above for 9 min on ice. Embryos were transferred to the SS mesh as a monolayer after CPA loading, with excess CPA solutions wicked away prior to plunge cooling. Images of embryos in liquid nitrogen were obtained to assess the ice formation after cooling (Fig. 4a). For 39% EG + 9% sorbitol and 33% EG + 9% sorbitol, we found that most embryos appeared transparent, indicating vitrification success. Note that the image shows ice flakes present in the upper right corner for 33% EG + 9% sorbitol, but it is expected that these were condensed from the ambient air, not inside the embryos (Fig. 4a). As CPA concentrations decreased to 27% EG + 9% sorbitol, the embryos started to appear hazy and some of them opaque (in the bottom area of the image), suggesting possible ice formation during cooling. In contrast, most of embryos turned opaque for 21% EG + 9% sorbitol, suggesting the presence of ice.

We next investigated embryo rewarming through numerical simulation of joule heating. The heat diffusion time across the embryo was ~1.3 ms. We selected the dimensions of SS mesh to be 15 cm long and 1 cm wide, which provided a resistance of 1.4 Ω and a RC time constant of 5.6 ms. To optimize the pulse width for joule heating, we simulated the temperature profile and warming rate of the embryos for 0.1 ms, 1 ms, and 10 ms pulses. The geometries of SS mesh and embryos used in the model are shown in Fig. 4b. We included two representative embryos with different patterns of contact with the heat source (i.e., the wires of SS mesh). We studied the temperature at the bottom of embryo (point C, next to the SS mesh), middle of embryo (point B), and top of the embryo (point A), as indicated in Fig. 4b. The temperature profiles illustrated an increasing temperature difference inside the embryos (i.e., point C vs point A) with decreasing pulse width (Supplementary Fig. 3). We selected a 1 ms pulse (comparable with *t*_*d*_ = 1.3 ms) to prioritize fast warming rates while avoiding large temperature gradients inside the embryo, compared with 0.1 ms and 10 ms pulses (Fig. 4c, Supplementary Fig. 3). As shown in Fig. 4d and Supplementary Fig. 3, the fraction of SS mesh that did not have a thermo load (i.e., no embryo contact, point E in Fig. 4b) had a higher temperature than its counterpart with a thermo load (i.e., embryo contact, point D in Fig. 4b). After the pulse ended, the SS mesh without embryo contact (i.e., Point E) continued to conduct heat toward the embryo, leading to a slightly higher warming rate around the periphery of the embryo (Fig. 4e). Figure 4e further demonstrated the similar distribution of warming rates between embryos with different contact with the SS mesh, as the aperture size of SS mesh (38 µm) was relatively small compared with the dimensions of an embryo (180 µm × 500 µm). This also suggests that mesh geometry will be an important factor to optimize for application in different biological system geometries.

To experimentally monitor the warming performance of joule heating, we employed a high-speed camera to detect the ice formation during rewarming. Embryos loaded with different CPA concentrations were rewarmed with 1 ms voltage pulse and recorded at 3000 frames per second (fps) under a dissecting microscope to capture more embryos. The grayscale intensity of embryos was analyzed using MATLAB. Higher intensity values were noted for white color (i.e., ice); lower intensity values (i.e., dark color) were noted when embryos were melted. For 27% EG + 9% sorbitol, images of embryos before and after joule heating are shown in Fig. 4f. Specifically, at *t* = 0 ms, embryos were partially vitrified prior to joule heating, which was applied at *t* = ~25 ms. At *t* = 50 ms, most embryos turned hazy, and a small amount of ice can be spotted, indicating that devitrification/recrystallization occurred during rewarming. The embryos appeared dark in color (i.e., similar to the SS mesh) at *t* = 150 ms, suggesting complete melting. In Fig. 4g, the grayscale intensities (i.e., normalized to *t* = 150 ms when embryos completely melted) for all four CPA concentrations were displayed. For 21% EG + 9% sorbitol and 27% EG + 9% sorbitol, after pulse heating, the intensity first increased and then decreased, indicating ice formation during rewarming. A higher peak was observed for the 21% EG group, suggesting more ice formation, as supported by the images in Supplementary Fig. 4. This is also expected as more ice was noted for 21% EG group after cooling (Fig. 4a). In contrast, for 33% EG + 9 % sorbitol and 39% EG + 9 % sorbitol, the intensity dropped after the pulse heating, implying the absence of apparent ice formation. The intensity of the 39% EG group dropped faster than that of the 33% EG group, which we speculate might imply a faster warming rate. Indeed, it took ~25 ms for the intensity to drop to a constant level for 39% EG group, which matched with the ~30 ms rewarming time (−196 °C to 0 °C) indicated by the numerical simulation (Fig. 4c).

The embryo survival was evaluated for different CPA concentrations and rewarming methods. The survival rates were reported as hatch rate and adult rate, which represent the embryo to larva survival and larva to adult survival, respectively. For CPA-treated embryos (i.e., no cryopreservation), we found that the hatch rate remained high (> 90%) across different CPA concentrations. However, the adult rate decreased with increasing CPA concentrations, suggesting CPA toxicity affected the larvae to adult development. For rewarming, to optimize the voltage for joule heating, we simulated the final temperature of the embryos, assuming ice-free rewarming for simplicity. Figure 4i indicated that voltages from 250 V to 290 V provided a final temperature ranging from −5 °C to 20 °C. In addition, the survival rates are displayed in Fig. 4j and Supplementary Fig. 5 for voltages from 230 V to 330 V at different CPA concentrations. The survival rates first increased then decreased with increasing voltages, transitioning from underheating to good warming and eventually overheating. For 39% EG group, no visible lethal ice was recorded during rewarming by 1 ms pulse joule heating (Fig. 4g) and high viability was observed (Fig. 4k), suggesting the temperature gradient during rewarming (Supplementary Fig. 3b) was tolerable in the *Drosophila* embryos. For the 27% EG group, although ice was observed during the rewarming (290 V, 1 ms pulse), as depicted in Fig. 4f, we showed that 60.8 ± 5.3% embryos hatched into larvae and 41.3 ± 6.9% larvae developed into adults. In contrast, for the 21% EG group, where more ice was identified during cooling (Fig. 4a), we found that the hatch rate and adult rate were 7.6 ± 3.5% and 6.7 ± 2.1%, respectively. At last, we compared the post-thaw survival rates between convective warming and joule heating, as shown in Fig. 4k. When 39% EG + 9% sorbitol was used, the convective warming produced similar survival with joule heating, suggesting that the convective warming rate may have already surpassed the CWR. Importantly, joule heating demonstrated higher survival than convective warming at lower CPA concentrations in our model system, which provides valuable opportunities for species less tolerant of the toxicity of high CPA concentrations.

### Joule heating for kidney slices cryopreservation

**Fig. 5:**
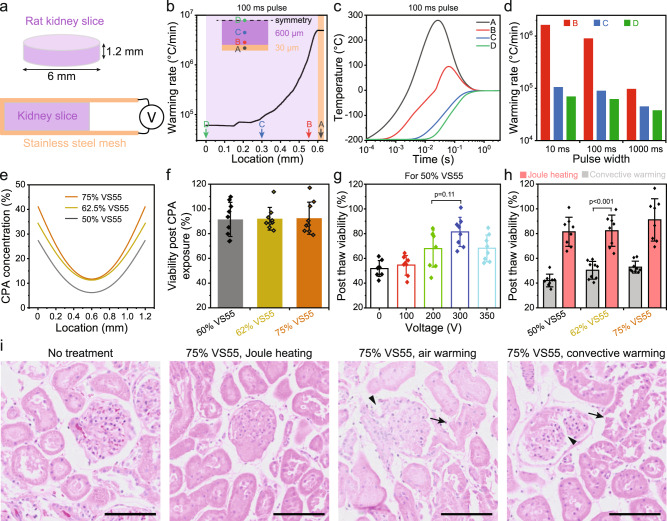
Kidney slice cryopreservation using joule heating. **a** Rat kidney slices of 1.2 mm thickness and 6 mm in diameter were used. After cryoprotective agent (CPA) loading, the kidney slice was sandwiched using the stainless steel (SS) mesh prior to plunge-cooling in liquid nitrogen, then rewarmed by connecting the SS mesh to a voltage source. **b** Warming rate distribution within the kidney slice. Point A represents the SS mesh; B, C, and D represent the kidney slice. The detailed locations of points A, B, C, and D were marked in the plot. **c** Temperature profile at different locations (point A to D) for 100 ms joule heating. **d** Warming rates at different locations within the kidney slice using 10 ms, 100 ms, and 1000 ms joule heating. **e** CPA concentration distribution within the kidney slice after loading with different CPA concentrations. **f** Viability of kidney slices after exposure to different CPA concentrations. *n* = 9 independent samples. **g** Post-thaw viability of the kidney slices by joule heating using different voltages. The CPA is 50% VS55. *n* = 8 independent samples. **h** Comparison of post-thaw viability of kidney slices using convective warming and joule heating for different CPA concentrations. *n* = 8 independent samples. **i** Histology (H & E staining) of kidney slices. Arrowhead showed disrupted glomeruli, and arrow indicated damaged proximal tubules. Scale bar is 500 µm. Modeling results were shown in **b**–**e**. For **f**–**h**, the viability was measured by alamarBlue assay and normalized by the readings prior to treatment. Date presented as mean ± s.d. One-way ANOVA and Tukey’s post hoc were used for statistical analysis.

With the successful implementation in sub-millimeter systems including adherent cells and *Drosophila* embryos, we further tested joule heating for cryopreservation of 1.2 mm thick rat kidney slices (diameter = 6 mm) as a model system. We used SS mesh to sandwich the kidney slice on each side to provide heating from both the top and bottom surfaces (Fig. 5a). Therefore, the biosystem heat diffusion distance was reduced to 0.6 mm, leading to a heat diffusion time of 180 ms. Numerical simulation was employed to optimize the pulse width (10 ms, 100 ms, and 1 s) for joule heating. Half of the kidney slice was modeled considering the symmetry (Fig. 5b). In Fig. 5b, we selected the center of the SS mesh (point A), and the bottom (point B), middle (point C), and top (point D) locations of the kidney slice for temperature and warming rate comparison. The temperature profiles of points A–D are displayed for different pulse widths in Supplementary Fig. 6. Based on this analysis, we selected a 100 ms pulse width to investigate further in the experiments. The warming rate across the thickness of the kidney slice is plotted in Fig. 5b. The temperature profile shown in Fig. 5c illustrates that the entire kidney slices took ~500 ms to reach an equilibrated final temperature while the pulse was 100 ms. The modeling suggests that the surface of the kidney slice (i.e., point B in Fig. 5c) could reach 40 to approximately 94 °C for a short period of time (30 ms to 170 ms) during rewarming. We used the Arrhenius model to estimate the potential for thermally induced injury and found negligible impact in viability (i.e., 3.2% decrease in viability, details in Methods).

We next optimized the joule heating voltage using modeling and viability assessment. CPA loading was performed in a stepwise fashion (10 min each step) on ice by passive diffusion to minimize osmotic shock. After loading, the CPA concentration inside the kidney slice was modeled and is shown in Fig. 5e. Due to the slow diffusion mechanism, the CPA concentration decreased towards the middle plane of kidney slices. For example, with 75% VS55, the CPA concentration loaded at the middle plane is 11.7 wt%, which further dropped to 6.2 wt% for 50% VS55. To ensure close contact for effective rewarming, a customized plastic tweezer with a 1.2 mm gap at the tip was used to hold the sandwich of the SS mesh/kidney slice during joule heating. We used alamarBlue to obtain fluorescence readings from the same kidney slice before and after cryopreservation. The viability was evaluated as the ratio of those two readings. For all three CPA concentrations, the viability was similar after CPA loading and unloading, as presented in Fig. 5f. To optimize the voltage for joule heating, we modeled the temperature profile. The predicted final temperature of the kidney slice after joule heating increased from −155, −83, and −1 to 32 °C for the voltages of 100 V, 200 V, 300 V, and 350 V, respectively (Supplementary Fig. 7). In addition, we examined the viability and showed that as the voltage increased, the viability increased until 300 V and dropped at 350 V (Fig. 5g and Supplementary Fig. 8). The decrease of viability at 350 V could be attributed to the overheating of the kidney slice surfaces (i.e., next to the SS mesh, Supplementary Fig. 7d) or increased CPA toxicity at the higher final temperature (i.e., 32 °C), or both. CPA exposure during cryopreservation protocols is typically conducted below at least 4 °C, as toxicity has generally been shown to increase substantially at higher temperatures.

Lastly, we compared the viability between conventional convective warming and joule heating including different dilutions (50%, 62.5%, 75%) of VS55 as the CPA. For the convective warming group, the CPA-loaded kidney slices were placed in a 2 ml cryovial, which was plunged into liquid nitrogen for cooling and convectively rewarmed using a water bath, as reported in the literature^18,20,41^. The convective warming rate was 100 to approximately 200 °C/min^20^. For direct comparison of SS mesh convective warming and joule heating, the kidney slices were sandwiched between the SS mesh, plunged into liquid nitrogen and then convectively rewarmed. The post-thaw viabilities for 50% VS55, 62.5% VS55, and 75% VS55 were 42.1 ± 4.8%, 50.3 ± 6.8%, and 52.9 ± 4.5% using cryovial convective warming; 54.9 ± 17%, 61 ± 12%, and 76.1 ± 18.2% using SS mesh convective warming (Supplementary Fig. 9); and 81.4 ± 11%, 82.3 ± 11.8%, and 91.1 ± 15.9% using SS mesh joule heating, respectively (Fig. 5h). In addition, to examine the gross structural morphology, we performed H&E histological staining of the conditions including a positive control (i.e., no treatment), optimal joule heating (300 V), air warming (i.e., negative control), and cryovial convective warming using 75% VS55 loaded kidney slices (Fig. 5i). For the joule heating group, intact structures of glomerulus can be identified, and the Bowman’s space was similar to the positive control group. In contrast, the convective warming groups showed disrupted glomeruli, and some proximal tubules were damaged. Notable architectural disruption of the glomerulus and pale-staining were observed for the air warming group. Overall, the viability measurement corroborated the histological results, demonstrating that joule heating showed great promise in improving cryopreservation of millimeter-scale thickness tissues using low CPA concentrations. More extensive examinations of tissue functionality will be important in the future technology development.

### Physical limits of joule heating platform technique

**Fig. 6:**
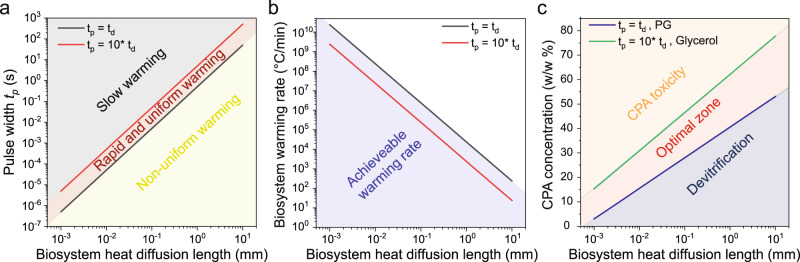
Design and physical limits of joule heating as the external heat source for rewarming. **a** To achieve rapid and sufficiently uniform rewarming (area in red), the pulse width (*t*_*p*_) can be selected based on biosystem heat diffusion length (*h*). When the pulse width is much smaller than heat diffusion time (*t*_*d*_ *= h*^2^*/2α*, *α* is the thermal diffusivity of the biosystem: 10^−6^ m^2^/s was used for the calculation), non-uniform warming occurs. For longer pulse widths (i.e., > 10 *t*_*d*_), the achieved rewarming rate is slow. We assume sufficiently uniform rewarming can be achieved using *t*_*p*_ = 1 ~ 10 *t*_*d*_. **b** Achievable warming rate inside the biosystems as a function of biosystem heat diffusion length. The black and red lines represent the warming rates (calculated as 200 °C */ t*_*p*_) when *t*_*p*_ *=* *t*_*d*_ and *t*_*p*_ *=* 10 *t*_*d*_, respectively. The achieved warming rate decreases with increasing pulse width. **c** Within the rapid and uniform warming region defined in **a**, selection of cryoprotective agent (CPA) concentration for optimal cryopreservation of different biosystems using external joule heating source. The upper bound of CPA concentration (green line) is estimated using the longer pulse width (i.e., *t*_*p*_ = 10 *t*_*d*_) and glycerol as the CPA (i.e., relatively high critical warming rate). The lower bound of CPA concentration (blue line) is estimated using the shorter pulse width (i.e., *t*_*p*_ *= t*_*d*_) and propylene glycol (PG) as the CPA (i.e., relatively low critical warming rate). The CPA toxicity is the major failure mode of cryopreservation in the upper corner region (i.e., orange color). The devitrification is the major failure mode of cryopreservation in the lower corner region (i.e., blue color).

We summarized the design principles and physical limits of joule heating for rewarming of biosystems with different heat diffusion lengths in Fig. 6. To design the joule heating for rapid rewarming of biosystems with minimal thermal gradient, we performed heat transfer scaling analysis. Based on the biosystem heat diffusion length (*h*), we estimated heat diffusion time (*t*_*d*_ = *h*^2^/2*α*) across the biosystems assuming heat diffusivity (*α*) of 10^−6^ m^2^/s for 2 M glycerol-laden tissues at sub-zero temperature^42^. As demonstrated in our modeling results, short pulse widths lead to non-uniform warming, and long pulse widths result in slow warming rates (Figs. 3a, 4c, 5d, Supplementary Figs. 3, 6). As a rule of thumb, the optimal range of pulse width is *t*_*d*_ to approximately 10 *t*_*d*_ to achieve rapid and sufficiently uniform warming. Specifically, the required input energy from joule heating to rewarm the biosystems remains similar regardless of the pulse width, assuming heat loss or gain from the ambient is negligible for short pulse widths (< 1 s). When *t*_*d*_ > *t*_*p*_, non-uniform warming occurs, as energy needs to be delivered within a short time period, causing a high temperature in the biosystem adjacent to the heat source, followed by heat diffusion to the entire biosystem after the voltage pulse ends. However, slow rewarming takes place for *t*_*d*_ ≪ *t*_*p*_. In this case, input energy is spread over a wide time window, limiting the warming rate but providing sufficient time for heat to diffuse across the entire biosystem for uniform warming. We did not observe cracking in all tested biosystems using optimal rewarming conditions and assume that pulse width of *t*_*d*_ ~10 *t*_*d*_ provided sufficiently uniform rewarming.

In addition, the achievable warming rate is limited by the biosystem heat diffusion length (Fig. 6b). The estimated overall warming rates can be calculated as 200 °C */ t*_*p*_, where 200 °C is the temperature difference from liquid nitrogen (−196 °C) to 4 °C. For example, when *t*_*p*_ = *t*_*d*_, estimated warming rates for biosystems with 10 µm, 100 µm, 1 mm, and 1 cm heat-diffusion lengths are 2 × 10^8^, 2 × 10^6^, 2 × 10^4^, and 2 × 10^2^ °C/min, respectively. With the achievable warming rates, the target CPA concentration inside the biosystems can be estimated to realize optimal cryopreservation (Fig. 6c). Two of the major failure modes of vitrification-based cryopreservation are CPA toxicity (i.e., CPA concentration too high) and devitrification (i.e., CPA concentration too low), assuming the biosystems can be successfully vitrified without cracking. Within the rapid and uniform warming region in Fig. 6a (i.e., *t*_*d*_ ≤ *t*_*p*_ ≤ *10 t*_*d*_), we estimated the upper and lower bounds of optimal CPA concentrations using a slower warming rate (*t*_*p*_ *= 10 t*_*d*_) with glycerol (relatively high CWR) as the CPA and a faster warming rate (*t*_*p*_ *= t*_*d*_) with PG (relatively low CWR) as the CPA, respectively. As a general guideline, we estimated that the lowest CPA concentrations (blue line in Fig. 6c) required for biosystems with 10 µm, 100 µm, 1 mm, and 1 cm heat diffusion lengths are 15%, 28%, 40%, and 53 w/w%, respectively. In practice, the optimal CPA concentration could be increased to facilitate successful vitrification given that CPA toxicity can be minimized with optimized loading and removal protocols.

## Discussion

We showed that a joule heating–based technique can achieve rewarming rates up to ~6 × 10^8^ °C/min for biosystems such as adherent cells, which represent the fastest rewarming rates reported in the cryopreservation field, to our best knowledge (Table 1). One major advantage of joule heating is the adjustable, wide range of warming rates enabled in one simple platform technique. Precision voltage pulses can be readily generated with microseconds to minutes or a longer pulse width. Guided by the scaling analysis and numerical modeling developed in this study, the voltage and pulse width can be tailored to achieve rapid warming in broad ranges of biosystems across multiple scales, thereby achieving lower CPA loading for reduced toxicity and the possibility to rescue from ice formation. In this study, we validated the performance of joule heating by comparing with convective warming, the gold standard method in the cryobiology field. Future systematic experimental comparisons with other warming methods listed in Table 1 will provide more insights into the capacity of the joule heating technique for specific scale systems or applications. In principle, as illustrated in Table 1 comparing the existing rewarming methods, joule heating provides the widest range of warming rates in a simple, scalable manner tailored to each biosystems at different scales. Additionally, similar to our recent laser calorimetry work^16^, joule heating can potentially be applied as a scanning calorimeter to investigate the CWR of intermediate CPA concentrations and readily cover a wider warming rate range. Due to the intrinsic heating mechanism, the joule heating technique provides close to 100% input energy conversion to heat. Energy loss occurs in the resistance of the circuit (< 0.1 Ω), which is usually a small portion. We noted that joule heating generated the highest SAR (up to ~6 × 10^13^ W/m^3^) compared to other external heating methods (Table 1). The heat source (i.e., electrical conductor) is reusable, cheap, and available in large scale. Further, the conductors used to deliver the energy can be designed (material and geometry) to match the specific requirements of a given biological system. Importantly, while this study focused on demonstration of feasibility in model biosystems, the joule heating technique could be scaled into channel or matrix constructs to rewarm from multiple directions and bulk volumes.

Regarding the concern about potential damage from electrical current flowing through the biosystems in this study, the electrical conductivity (*σ*) of cells and tissues are commonly on the order of 1 S/m or lower^43–45^. Therefore, the majority of the electrical current flows through the SS (*σ* ~3 × 10^5^ S/m). A thin, thermally conductive, dielectric layer could be coated on the conductor (similar to the commercial thin film resistor) to eliminate this concern. Another important aspect is whether the joule heating is uniform within the conductor during a short voltage pulse. When alternating electric current (AC) passes through a conductor, the fast change of electrical field could lead to an additional internal induction field, resulting in higher current densities toward the surface of the conductor. Consequently, the electrical current flows mainly within the skin depth from the outer surface of the conductor, increasing the resistance and causing inhomogeneous joule heating within the conductor (i.e., higher heating at the outer surface)^21^. The skin depth (*δ*) can be calculated as shown in Eq. (1):

1 $$\delta=\sqrt{1/\pi f\sigma \mu }$$

where *f* is the frequency, *σ* is the electrical conductivity of the conductor, and $\mu$ is the magnetic permeability of the conductor. Similarly, for the capacitive discharge using SS in this study, where *f* is ~1/pulse width, *σ* is 3.57 × 10^5^ S/m, and $\mu$ is 1.26 × 10^˗6^ H/m, we estimated the skin depth to be 2.7 mm for a 10 µs pulse, which is two orders of magnitude larger than the thickness (12 µm) of SS sheet used for adherent cell cryopreservation, indicating uniform heat generation within the SS sheet. Considering the skin depth increases with pulse width (10 µs to 100 ms in this study), the current densities and joule heating within the SS sheet (12 µm thickness) and mesh (30 µm thickness) are homogeneous. Further, even in cases where these effects may take place, thermal conduction within the heating element will dominate conduction into the biological system, so the heater will essentially equilibrate on the timescales of conduction within the biological system.

In addition, we showed direct videographic evidence to prove that certain small amounts of ice formation during rewarming are not lethal when rapid rewarming is applied. Specifically, *Drosophila* embryos treated with 27% EG + 9% sorbitol were partially vitrified after cooling, rewarmed with 1 ms pulse at ~4.2 × 10^6^ °C/min and recorded at 3000 fps. Ice formation was observed after voltage pulse and lasted ~30 ms inside the embryos (Fig. 4f, g, Supplementary Movie 1). Notably, we demonstrated that 60.8 ± 5.3% embryos hatched into larvae and 41.3 ± 6.9% larvae developed into adults. In comparison, with the similar warming rate, the survival decreased sharply when a lower CPA concentration (21% EG + 9% sorbitol) was used and more ice observed during rewarming (Fig. 5g, k, Supplementary Fig. 4, Supplementary Movie 2). The amount of tolerable ice will likely depend greatly on the biological system and where the ice forms in the system; however, this effect has been previously hypothesized in the literature^46–49^.

Our work opens up exciting opportunities for future directions: (1) The rapid warming rates generated by joule heating could help to lower the CPA concentration and reduce toxicity, improving the cryopreservation of numerous biosystems that are intolerable to the toxicity of high CPA concentrations, such as aquatic species and mosquito larvae and embryos. (2) Scaling up for high throughput cryopreservation. With almost 100% of input energy converted into thermal energy for rewarming, joule heating is a scalable platform technique. For example, a 290 V, 1 ms voltage pulse was used to rewarm a 15 cm × 1 cm SS mesh (i.e., 1.4 Ω) loaded with *Drosophila* embryos in this study. To scale up the area of SS mesh by 9-fold (i.e., 45 cm × 3 cm), the resistance remains the same and the voltage only needs to increase by 3-fold (i.e., 870 V) to achieve the same warming rates (Eqs. (2) and (7)). Therefore, joule heating would allow high throughput cryopreservation while maintaining the rapid warming rates, which could provide profound implications for numerous important biosystems such as coral species. Further, this can be achieved with basic engineering materials and modification to existing pulse generator technologies. (3) Spatial-temporal control of warming rate can be further achieved by electrical and material designs. In our study, we employed simple 2D materials such as a SS mesh and sheet and regular lab equipment such as the pulse generator in order to investigate different pulse widths. Depending on the biosystem heat diffusion length, we showed that the applied pulse parameters can be optimized for each biosystem application to achieve rapid rewarming. Therefore, a much cheaper and simpler capacitive discharge setup could be prepared for a specific biosystem. The exponentially decaying voltage pulse used in this study can also be replaced with a square wave or other forms of voltage pulse for controlled temporal warming rates. Lastly, various materials in different form factor designs could extend the joule heating to achieve controlled spatial warming rates for cryopreservation of planar or radial systems such as cartilage, blood vessels, and more complex tissues. In these cases, the ability to warm from two sides or from a boundary inward (i.e., radial system) would in principle allow one to achieve higher warming rates as needed to avoid crystallization failure in lower CPA-loaded tissue (i.e., away from the boundary).

## Methods

### Animals

All animal protocols were approved by the Institutional Animal Care and Use Committee (IACUC) at the University of Minnesota (IACUC Protocol: 1905-37029A).

### Voltage, current and resistance measurement

A commercial pulse generator (ECM® 830, BTX® Harvard Apparatus) was used in this study. The low voltage (LV, voltage ≤ 500 V) mode was used with the adjustable voltage and pulse width. Stainless steel (SS) 304 was chosen for the heating material. For the SS sheet, the thickness was 12 µm. For the SS mesh, the wire diameter was 30 µm, and aperture (i.e., pore size) was 38 µm. The current and voltage were measured by a current probe (P6021A, Tektronix) and voltage probe (Enhancer 3000, Harvard Apparatus), respectively. An oscilloscope (DS1M12, USB Instrument) was used to record the measured current and voltage. The sample rate of this oscilloscope is up to 1 MS/s (i.e., 1 × 10^6^ samples per second). The bandwidth of this oscilloscope is 250 kHz. For non-sinusoidal waveforms (i.e., square waves, pulses, etc.), a bandwidth of 10 times the fundamental frequency (1/pulse width in our case) is appropriate to capture the rise of signal. Therefore, for pulse widths larger than 40 µs, this oscilloscope provides reliable measurements. To accurately measure the resistance of a SS sheet and mesh with certain dimensions, the voltage was altered to establish the voltage-current correlation where the resistance can be calculated. The effective electrical conductivity (*σ*) of SS mesh or sheet was calculated based on the dimensions and measured resistance.

### Temperature measurement of commercial thin film resistor

Commercial thin film resistors (150 Ω) with electrically insulated surface coating were used to measure the temperature during joule heating. An ultrafine gauge type T thermocouple (COCO-002, OMEGA) was attached to the surface of a resistor using scotch tape to ensure close contact. No aqueous solution was involved during this measurement. The temperature was recorded using an oscilloscope (DS1M12, USB Instrument). The resistor was submerged in liquid nitrogen during the entire measurement process. One electric pulse (5 ms) was applied, and the temperature was recorded at a resolution of 1 ms. Warming rate was calculated from the temperature zone −170 °C to −10 °C in this study unless otherwise noted. Different voltages ranging from 100 V to 300 V were tested. Note that when a thermocouple was directly in contact with SS sheet or mesh, the applied voltage pulse interfered with the thermocouple signal. Therefore, we did not directly measure the temperature of SS sheet or mesh.

### Energy delivery and specific absorption rate (SAR)

A single pulse was used in this study. For the low voltage mode of the pulse generator, the capacitance is 4 mF, maximum current is 500 A, maximum voltage is 500 V, and the pulse width range is 10 µs to approximately 10 s. The thermal energy generated by the joule heating per pulse can be calculated as below:

2 $$Q={\int }_{0}^{{pw}}\frac{{U}^{2}}{R}{dt}={\int }_{0}^{{pw}}\frac{{({U}_{0}{e}^{-t/{RC}})}^{2}}{R}{dt}=\frac{C{{U}_{0}}^{2}}{2}(1-{e}^{-\frac{2{pw}}{{RC}}})$$

Or equivalently:

3 $$Q={\int }_{0}^{{pw}}{I}^{2}{Rdt}={\int }_{0}^{{pw}}({I}_{0}{e}^{-t/{RC}})^{2}{Rdt}=\frac{C{R}^{2}{{I}_{0}}^{2}}{2}(1-{e}^{-\frac{2{pw}}{{RC}}})$$

where *Q* is the energy generated by joule heating, *R* is the resistance of the electrical conductor in contact with the biosystems, *C* is the capacitance of the capacitor, *U* is voltage, *I* is current, *U*_*0*_ and *I*_*0*_ are the peak voltage and current, *t* is time, and *pw* is pulse width. For simplicity, *R* is considered a constant in Eqs. (2), (3).

To calculate the maximum energy delivered per pulse shown in Fig. 2g, Eq. (2) was used for *R* ≥ 1 Ω and *U*_*0*_ set to 500 V; Eq. (3) was used for *R* ≤ 1 Ω and *I*_*0*_ set to 500 A.

To estimate the warming rate of the electrical conductor, the temperature change within the pulse width can be calculated as below:

4 $$Q=\frac{C{R}^{2}{{I}_{0}}^{2}}{2}\left(1-{e}^{-\frac{2{pw}}{{RC}}}\right)=m{C}_{p}\triangle T$$

5 $$R=\frac{L}{\sigma A}$$

6 $$m=\rho {LA}$$

7 $$\triangle T=\frac{C{{I}_{0}}^{2}}{2}\left(1-{e}^{-\frac{2{pw}}{{RC}}}\right)\frac{{R}^{2}}{m{C}_{p}}\propto \frac{{R}^{2}}{m{C}_{p}}=\frac{L}{{A}^{3}}\frac{1}{{\sigma }^{2}\rho {C}_{p}}$$

where, for the electrical conductor, *m* is mass, *ρ* is density, *L* is length, *A* is cross section area, *σ* is electrical conductivity, *C*_*p*_ is heat capacity, *ΔT* is the temperature change, as shown in Fig. 2d.

The specific absorption rate (SAR, W/m^3^), the heat source term used in the heat transfer modeling, is estimated as shown below:

8 $${SAR}=\frac{{({U}_{0}{e}^{-t/{RC}})}^{2}}{{RV}}$$

9 $$R={R}_{0}(1+\alpha (T-{T}_{0}))$$

where *V* is the volume of the electrical conductor, *R*_*0*_ is the resistance at 293 K, *T*_*0*_ is 293 K, and *α* is the temperature coefficient of resistivity (0.94 × 10^−3^ K^−1^ for stainless steel).

### Adherent cells cryopreservation

Human dermal fibroblasts (HDFs) were cultured in Dulbecco’s modified Eagle media (DMEM), supplemented with 10% fetal bovine serum (Thermo Fisher Scientific) and 1% penicillin-streptomycin (Sigma) at 37 °C under 5% CO_2_. The HDFs were purchased from ATCC (PCS-201-012) and stored in a liquid nitrogen Dewar flask maintained in the lab. Prior to adding HDFs, the SS sheet was sterilized by ethanol spray and UV irradiation. After adding cells to the SS sheet, overnight incubation was allowed for cell attachment before cryopreservation.

For cryopreservation, various dilutions (25%, 50%, 75%, and 100%) of VS55 were used as the CPA. CPA loading and unloading were performed stepwise with 3 min for each step at 4 °C^38^. The CPA concentration for each loading and unloading step was 18.75%, 25%, 50%, 75%, and 100% VS55. At the end of CPA loading, the excess CPA solution on the SS sheet was removed. The SS sheet with attached cells was plunged into liquid nitrogen for cooling. For convective warming, the SS sheet was plunged into the unloading solution at 4 °C, followed by stepwise CPA unloading. For joule heating, the SS sheet was connected to the pulse generator, then brought out of liquid nitrogen and immediately rewarmed by the voltage pulse, followed by stepwise CPA unloading. Using a stopwatch, we measured that it took 0.25-0.4 s to bring out the SS sheet from liquid nitrogen until the application of joule heating pulse. Assuming a convective heat transfer coefficient of 10 W/m^2^/K in air, heat transfer modeling suggested that the temperature of cells increased to −173~−161 °C (Supplementary Fig. 10), still below the glass transition temperature. The tested pulse widths included 10 µs, 100 µs, and 1 ms.

To measure the convective cooling and warming rates using the SS sheet, an ultrafine gauge type T thermocouple (COCO-002, OMEGA) was attached to the surface of the SS sheet. The temperature was recorded using an oscilloscope (DS1M12, USB Instrument) when the SS sheet was plunged into liquid nitrogen for cooling and into a water bath for rewarming.

Hoechst and propidium iodide (PI) were used to assess cell viability by membrane integrity. Fluorescent microscope images were taken by an Olympus IX50 microscope using 4x magnification. Fiji (ImageJ 1.52a) was used to quantify the viability.

### *Drosophila melanogaster* embryos cryopreservation

A wildtype stock (named WC1b, as previously reported^17^) of *Drosophila melanogaster* was used in this study. The flies were maintained at room temperature. Following the previously reported protocol, embryos were collected in a 1 h period on a grape juice plate smeared with yeast paste and incubated in a 20 °C incubator until reaching 22 h old. The embryos were then dechorionated and permeabilized for CPA loading. The first CPA loading step involved incubation in 13% EG at room temperature for 25 min. Next, the embryos were transferred to various dehydration CPAs on ice for 9 min^17^. In this study, four different dehydration CPAs were tested including 21% EG + 9% sorbitol, 27% EG + 9% sorbitol, 33% EG + 9% sorbitol, and 39% EG + 9% sorbitol. The dehydrated embryos were transferred to the SS mesh, and excess CPA solution was wicked away. The SS mesh with the embryos were quickly plunged into liquid nitrogen. The embryos remain attached to the SS mesh in liquid nitrogen due to the presence of residual CPA solution between the embryo and SS mesh. To confirm whether the embryos were vitrified, the S.S mesh with embryos were placed in liquid nitrogen vapor and observed under a dissecting microscope. Images of embryos in liquid nitrogen were taken using a C-mount microscope camera (MU1000, AmScope).

For convective warming, the SS mesh was rapidly plunged into 30% sucrose solution. After a few seconds, the SS mesh and embryos were transferred to 15% sucrose for 3 min, followed by washing in the isotonic buffer for 20 min for CPA unloading. For joule heating, the SS mesh was connected to the pulse generator, brought out of liquid nitrogen, and immediately rewarmed by a voltage pulse. The tested pulse width was 1 ms. The embryos were placed in 15% sucrose for 3 min and isotonic buffer for 20 min for CPA removal.

After CPA removal, the embryos were transferred to a 35 mm petri dish with 1 ml Schneider medium for further development. On the next day, the hatched larvae were counted and transferred to the food vials (15 × 95 mm shell vial) for development into adults. The hatch rate (i.e., embryo to larva rate) was calculated using the ratio of hatched larvae to total number of embryos. After 15 days at room temperature, the adult rate (i.e., larva to adult) was calculated using the ratio of emerged adults to total number of larvae placed in the vials.

### Joule heating high-speed video of *Drosophila* embryos

To observe ice formation during the rapid joule heating process, a high-speed camera (MEMRECAM Q1v, nac Image Technology) was used to record the rewarming of *Drosophila* embryos on SS mesh at 3000 frames per second (fps). *Drosophila* embryos were selected because (1) the frame rate of the high-speed camera was not fast enough to capture the rewarming of HDF cells (a 10 µs pulse was used for rewarming) and (2) the thickness of the inside of kidney slices (1.2 mm) made them challenging to visualize. To capture more embryos, the high-speed camera was mounted to a dissecting microscope. Four different dehydration CPAs including 21% EG + 9% sorbitol, 27% EG + 9% sorbitol, 33% EG + 9% sorbitol, and 39% EG + 9% sorbitol were tested. A customized MATLAB script was used to extract the time-dependent grayscale intensity of embryos during the joule heating process.

### Kidney slices cryopreservation

Kidneys were procured from 3-month-old Sprague-Dawley rats, following procedural protocol approved by Institutional Animal Care and Use Committee (IACUC) at the University of Minnesota (IACUC Protocol: 1905-37029A). Heparin (1000 IU) was injected intraperitoneally to the rat, 1–2 min before euthanasia. Immediately after confirmed death by exsanguination, the kidneys were collected by cutting open the abdomen. The kidneys were rinsed briefly with PBS to remove the blood and placed in UW solution for transportation. Kidney cortex slices with 1.2 mm thickness were prepared using the custom-modified Stadie-Riggs tissue slicer. The slices were incubated with 10% alamarBlue at 37 °C for 2 h to obtain the fluorescent readings before treatment. Three final CPA concentrations (50% VS55, 62.5% VS55, and 75% VS55) were tested. CPA loading was performed on ice in a stepwise manner with 10 min step duration, as previously reported^21^. At the end of CPA loading, the kidney slice was sandwiched by a pre-folded SS mesh strip, and the excess CPA solution was removed. Light plastic forceps were modified such that the gap at the tip of the forceps was 1.2 mm. The modified forceps were used to hold the SS mesh and kidney slice, maintaining close contact between them when plunged into liquid nitrogen. The forceps were removed after cooling. To rewarm the sample, the SS mesh was first connected to the pulse generator and brought out of liquid nitrogen. Before applying the voltage pulse, modified plastic forceps at room temperature were used to hold the SS mesh and kidney slice to ensure close contact during joule heating. After rewarming, the tissue was subjected to stepwise CPA unloading on ice. For SS mesh convective warming, the kidney slice was sandwiches between SS mesh, plunged into liquid nitrogen and convective rewarmed.

The cryovial convective warming was performed using a cryovial as reported in the literature^18^. Briefly, after CPA loading, the kidney slice was transferred to a cryovial with 1 ml final concentration CPA solution. The cryovial was plunged into liquid nitrogen for cooling and plunged into room temperature water bath (~2 min) for rewarming.

Viability of kidney slices was assessed by incubating the tissue with 10% alamarBlue at 37 °C for 2 h. This 2 h incubation provided enough time for alamarBlue to penetrate the 1.2 mm tissue, as suggested by a previous study^21^. A plate reader (Synergy HT, BioTek) was used to read the fluorescence of the supernatant. Data was recorded with Biotek Gen5 software. Kidney slices were incubated with alamarBlue before and after cryopreservation to obtain the corresponding fluorescence readings. The viability was reported as the ratio of those two readings from the same slice to avoid sample-to-sample variations. For the assessment of tissue viability, alamar blue (i.e., resazurin) is reduced to a fluorescent derivative, resorufin, following incubation with cells/ tissues. Resorufin fluorescence is extracted from the cells into the medium and the fluorescence of the supernatant can be monitored as a proxy for viability. Alamar blue has the advantages of low toxicity, high sensitivity, simplicity-of-use and extensive usage in the literature. The disadvantages include the semiquantitative nature of the assay and dependence on the metabolic pathway which can be affected by cellular status.

Histology was performed on kidney slices from various treatment conditions including positive control (no treatment), joule heating (290 V, 100 ms) of 75% VS55 loaded kidney slices, air warming of 75% VS55 loaded kidney slices, and convective warming of 75% loaded kidney slices. Kidney slices were transferred to 10% neutral buffered formalin and paraffin embedded within 48 h. Sections of ~5 µm thickness were used for hematoxylin and eosin (H & E) staining and imaging.

### Heat transfer modeling

The heat transfer was modeled using COMSOL 5.5 for the joule heating of adherent cells, *Drosophila* embryos, and kidney slices. The governing equations for the SS and biosystems are given as follow:

10 $${\rho }_{s}{C}_{p{{{{{\rm{\_}}}}}}S}\frac{\partial {T}_{S}}{\partial t}={k}_{S}{\nabla }^{2}{T}_{S}+{SAR}$$

11 $${\rho }_{B}{C}_{p{{{{{\rm{\_}}}}}}B}\frac{\partial {T}_{B}}{\partial t}={k}_{B}{\nabla }^{2}{T}_{B}$$

The subscript *S* represents SS sheet or mesh, subscript *B* represents biosystem, *SAR* is calculated from Eqs. (8), (9), *ρ* is density, *C*_*p*_ is heat capacity, *k* is thermal conductivity, and *T* is temperature. For various biosystems (adherent cells, *Drosophila* embryos, kidney slices), in the absence of experimental data on the material properties, we used the thermal properties of relevant samples as previously reported in the literature. Specifically, temperature dependent density of VS55^50^ was used for all biosystems. The reported temperature dependent thermal conductivity of 6 M glycerol-laden porcine liver^42^ was used as the estimation for all biosystems. For heat capacity, similar thermal properties have been reported between CPA solutions and CPA-loaded biological samples^21,25,50^, we used the experimentally measured values for VS55^25^ as the approximation for all biosystems. More detailed characterization of the thermal properties for CPA-laden biosystems (cells, embryos and tissues) is needed in the field, but our simulation should provide an initial approximation of the heat transfer process. For the SS, density was assumed to be constant (7.8 × 10^3^ kg/m^3^), and the temperature-dependent thermal conductivity and specific heat were obtained from National Institute of Standards and Technology (https://trc.nist.gov/cryogenics/materials/304Stainless/304Stainless_rev.htm).

The boundary condition for outer surfaces was natural convection at room temperature (293 K). The convective heat transfer coefficient (*h*_*conv*_) was assumed to be 10 W/m^2^/K. For the adherent cell modeling, the thickness of the cell monolayer was set to 4 µm, as reported in the literature^51^. The thickness of *Drosophila* embryos was 50 µm as previously reported^17^.

For different biosystems, we selected the geometry to capture the key dimensions affecting the heat transfer and minimize the cost of computational resources. For adherent cells, a 2D area (50 µm width, capturing the thickness of the cell monolayer and S.S. sheet, Supplementary Fig. 11) from the cross section perpendicular to the length of S.S. sheet was used. After CPA dehydration, the thickness of the *Drosophila* embryo was reduced to 50 µm. A 3D geometry (Supplementary Fig. 11) was used as the sizes of a *Drosophila* embryo (500 * 180 * 50 µm) and S.S mesh (wire diameter 30 µm, aperture size 38 µm) are on the same order of magnitude. For kidney slides, a 2D area (1 mm width, Supplementary Fig. 11) from the cross section perpendicular to the diameter of the kidney slices was used. The detailed dimensions and computational meshing (i.e., finite element method) can be found in Supplementary Fig. 11.

### Kidney slices CPA loading and thermal injury modeling

The Fick’s second law shown below was used to model the CPA diffusion into the kidney slices:

12 $$\frac{1}{D}\frac{\partial C}{\partial t}=\frac{{\partial }^{2}C}{\partial {x}^{2}}$$

*C* is CPA concentration, *t* is time, *D* is the effective diffusion coefficient (assumed to be concentration independent for simplicity), and x is the location inside the tissue. We used *D* = 6.5 × 10^˗11^ m^2^/s, as experimentally measured for the loading of VS55 into tissues in a previous study^21^. We used COMSOL 5.5 for the numerical simulation. Protocols with different final CPA concentrations, including 50% VS55, 62.5% VS55, and 75% VS55, were modeled. The distributions of CPA concentration after loading were plotted.

To assess the potential thermal injury of kidney slice due to overheating during joule heating (i.e., point B in Fig. 5c), the resultant thermally induced injury can be estimated assuming a first order kinetic process using Arrhenius model:

13 $$S=1-{e}^{-{\int }_{0}^{t}A{e}^{-(\frac{\triangle E}{{RT}})}{dt}}$$

where *S* is the thermally induced injury (i.e., drop in viability), *ΔE* is activation energy, *A* is frequency factor, *R* is universal gas constant (8.314 J/mole/K), and *T* is temperature (in Kelvin). Activation energy was 168.42 kJ/mole and the frequency factor was 2.53 × 10^24^ s^−1^, obtained from the literature^52^.

### Statistics and reproducibility

Experimental data was presented as mean ± s.d. unless otherwise specified. Sample size (n) for experimental data was included in the figure captions. For Figs. 3f, 4a, representative images were shown from *n* = 4 independent experiments with similar results. For plots with two dependent variables, hatch rate and adult rate for *Drosophila* embryo experiments, multivariate analysis of variance (MANOVA), and Tukey’s post hoc test were used for statistical analysis. A paired one-way ANOVA and Tukey’s post hoc test were used for statistical analysis of other experimental results using Graphpad8. The *p*-values > 0.05 were considered statistically non-significant (ns). The *p*-values < 0.05 were considered statistically significant, with *p < 0.05, **p < 0.01, and ***p < 0.001.

### Reporting summary

Further information on research design is available in the Nature Research Reporting Summary linked to this article.

## Supplementary information

Supplementary Information

Peer Review File

Description of Additional Supplementary Files

Supplementary Movie 1

Supplementary Movie 2

Reporting Summary

## Data Availability

All data are included in the main text and supplementary materials. Source data is available for Figs. 2–6 in the associated source data file. Source data are provided with this paper.

## Source data

Source Data

## Code availability

MATLAB 2019a was used to process the *Drosophila* embryos’ joule heating high-speed videos. COMSOL 5.5 was used for the heat transfer modeling. The customized codes are freely available from Zenodo (10.5281/zenodo.7054430).
