# Supplementary material for: Rapid joule heating improves vitrification based cryopreservation
Source: Nat Commun. 2022 Oct 12;13:6017. doi: 10.1038/s41467-022-33546-9 (PMC9556611; doi:10.1038/s41467-022-33546-9)
Supplement: Supplementary file 1 — Supplementary Information [file 41467_2022_33546_MOESM1_ESM.pdf]

Supplementary Information

Rapid joule heating improves vitrification based cryopreservation

Li Zhan[1*†], Zonghu Han[1], Qi Shao[1], Michael Etheridge[1], Thomas Hays[2], John Bischof[1,3*]

[1] Department of Mechanical Engineering, University of Minnesota, Minneapolis, MN, USA

[2] Department of Genetics, Cell Biology and Development, University of Minnesota, Minneapolis, MN, USA

[3] Department of Biomedical Engineering, University of Minnesota, Minneapolis, MN, USA

[†] Present address: Center for Engineering in Medicine, Massachusetts General Hospital, Shriners Hospital for Children, Harvard Medical School, Boston, MA, USA

* To whom correspondence may be addressed to. E-mail: lzhan@mgh.harvard.edu ; bischof@umn.edu

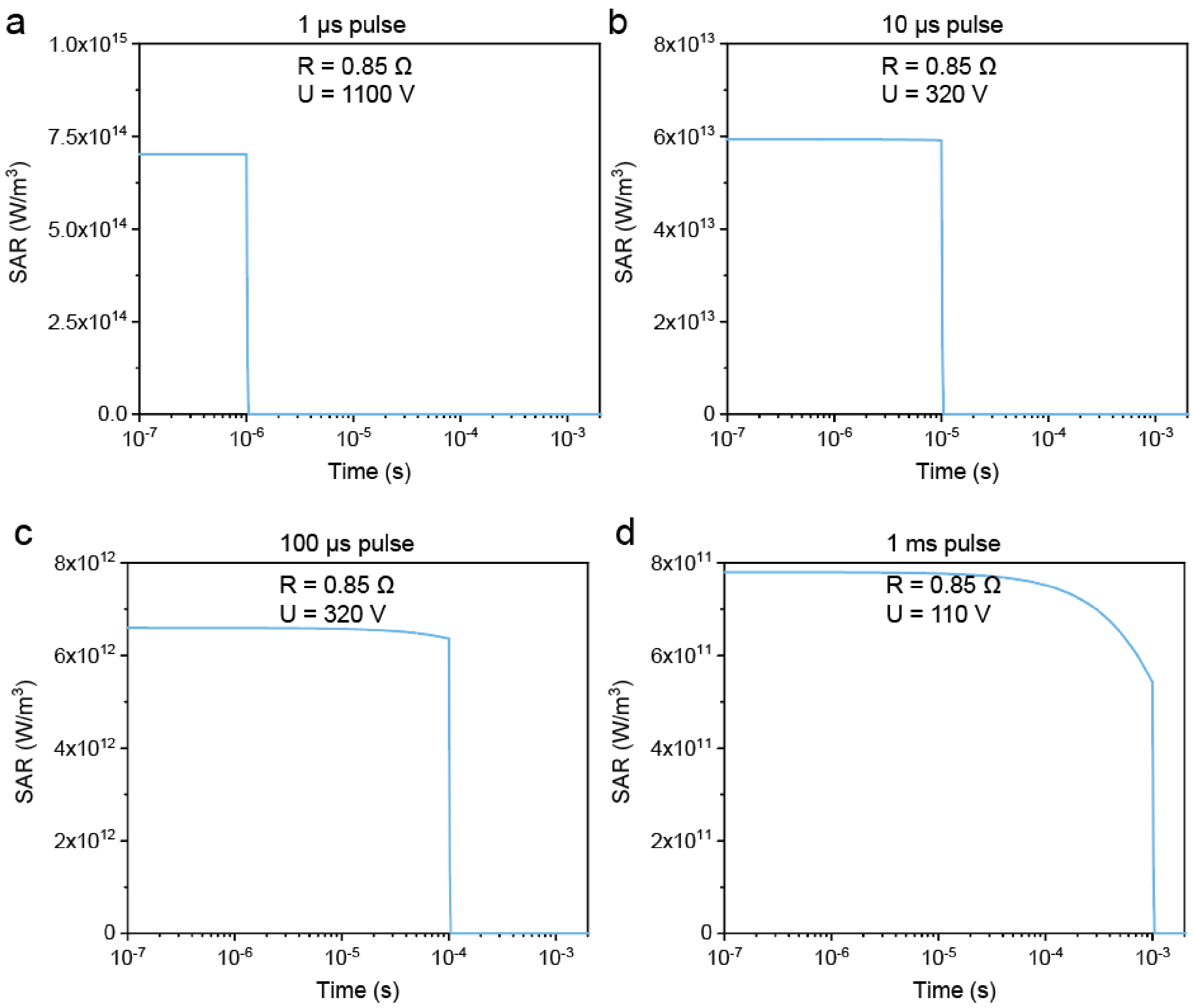

Supplementary Fig.1 Specific absorption rate (SAR) of the stainless steel (SS) sheet joule heating for adherent cell cryopreservation. The SAR profiles of 1 μs (a), 10 μs (b), 100 μs (c) and 1 ms (d) voltage pulse were used to model the temperature profile shown in Figure 3a. The resistance and voltage values were shown in the plot.

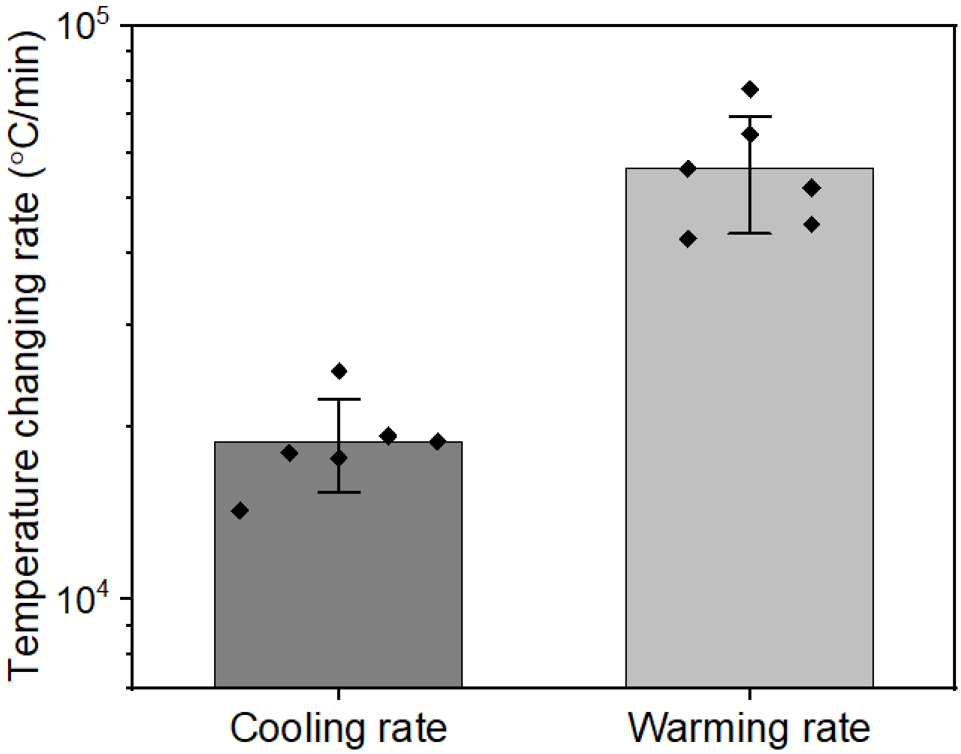

Supplementary Fig.2 The measured convective cooling and warming rate of stainless steel (SS) sheet for adherent cells cryopreservation. n=6 independent experiments. Data are presented as mean values ± s.d.

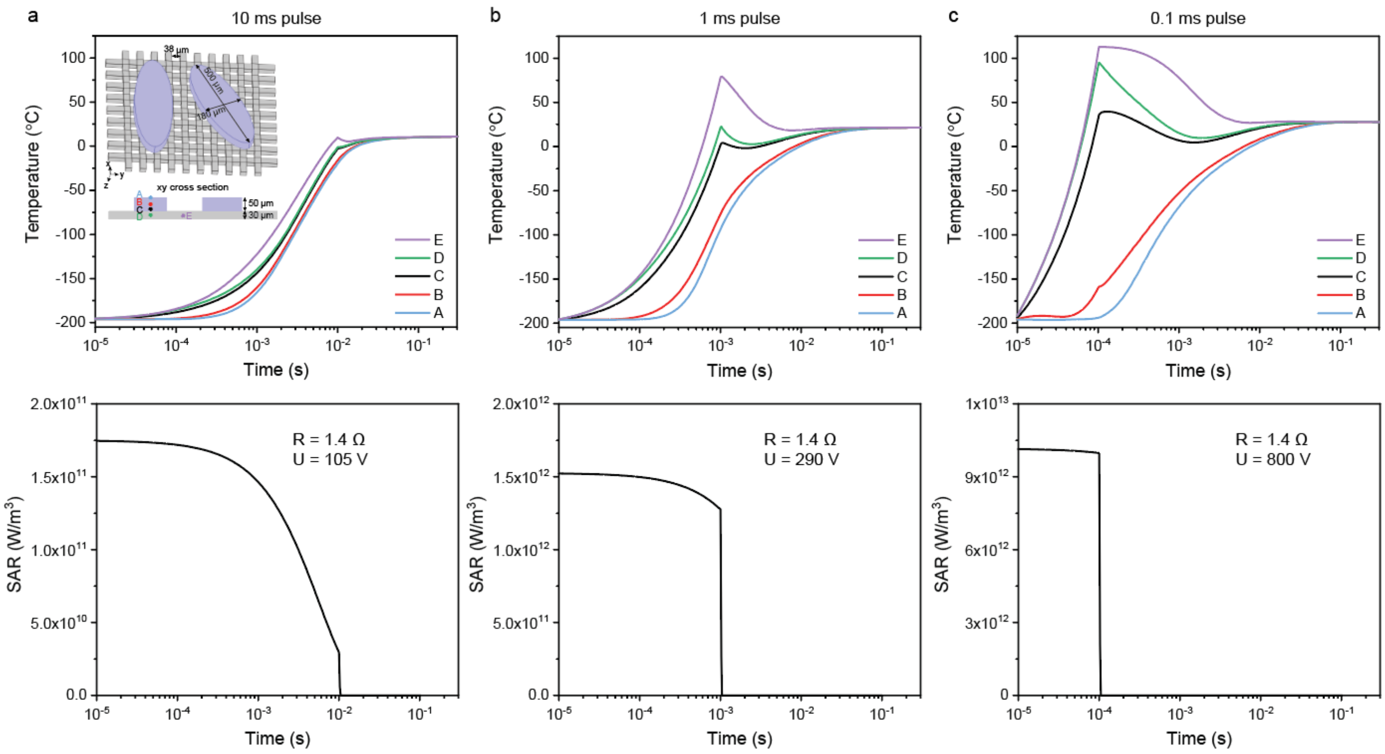

Supplementary Fig.3 The simulated temperature and specific absorption rate (SAR) profiles of the stainless steel (SS) mesh joule heating for *Drosophila* embryos cryopreservation. The SAR profiles (lower panel) of 10 ms (a), 1 ms (b), and 0.1 ms (c) voltage pulse were used to model the temperature profiles (upper panel). Points A, B, and C represent the top, middle and bottom of the embryo, respectively. Point D represents the SS mesh in contact with the embryo. Point E represents the SS mesh outside the embryo. The resistance and voltage values were shown in the plot.

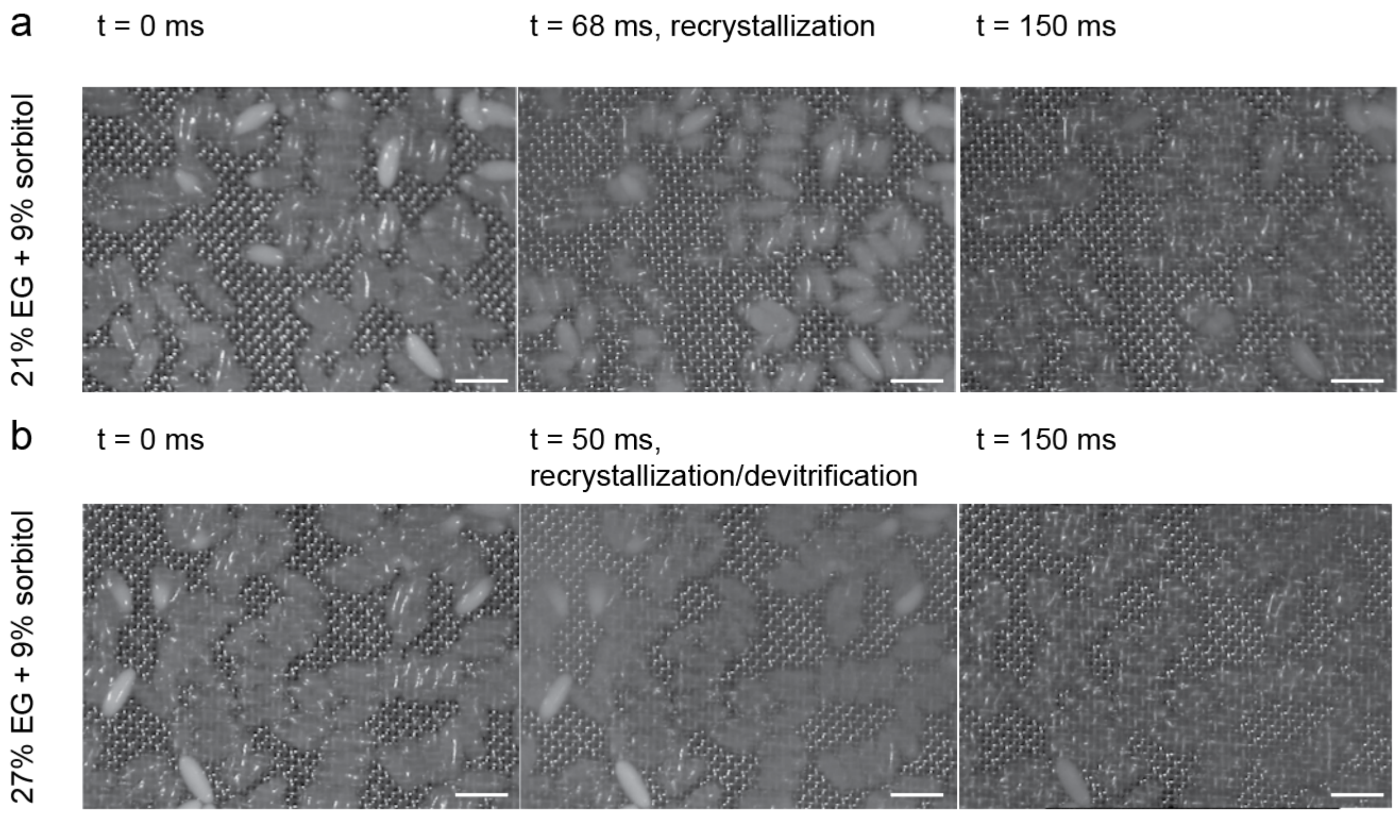

Supplementary Fig.4 Images of *Drosophila* embryos on the stainless steel (SS) mesh acquired by a high-speed camera at 3000 fps. For both 21% EG + 9% sorbitol (a) and 27% EG + 9% sorbitol (b), after 1 ms pulse at 290 V joule heating (applied at t = ~25 ms). In case a, the embryos first appeared white (i.e., recrystallization/devitrification at t = 68 ms), followed by the melting of ice at t = 150 ms. In case b., with 6% additional EG shows a lower degree of ice formation (i.e., less intense white and more transparent – i.e., vitrified embryos). The normalized grayscale intensity of the embryos was shown in Figure 4g. Scale bar is 500 µm. Representative images were shown from n=5 independent experiments with similar results.

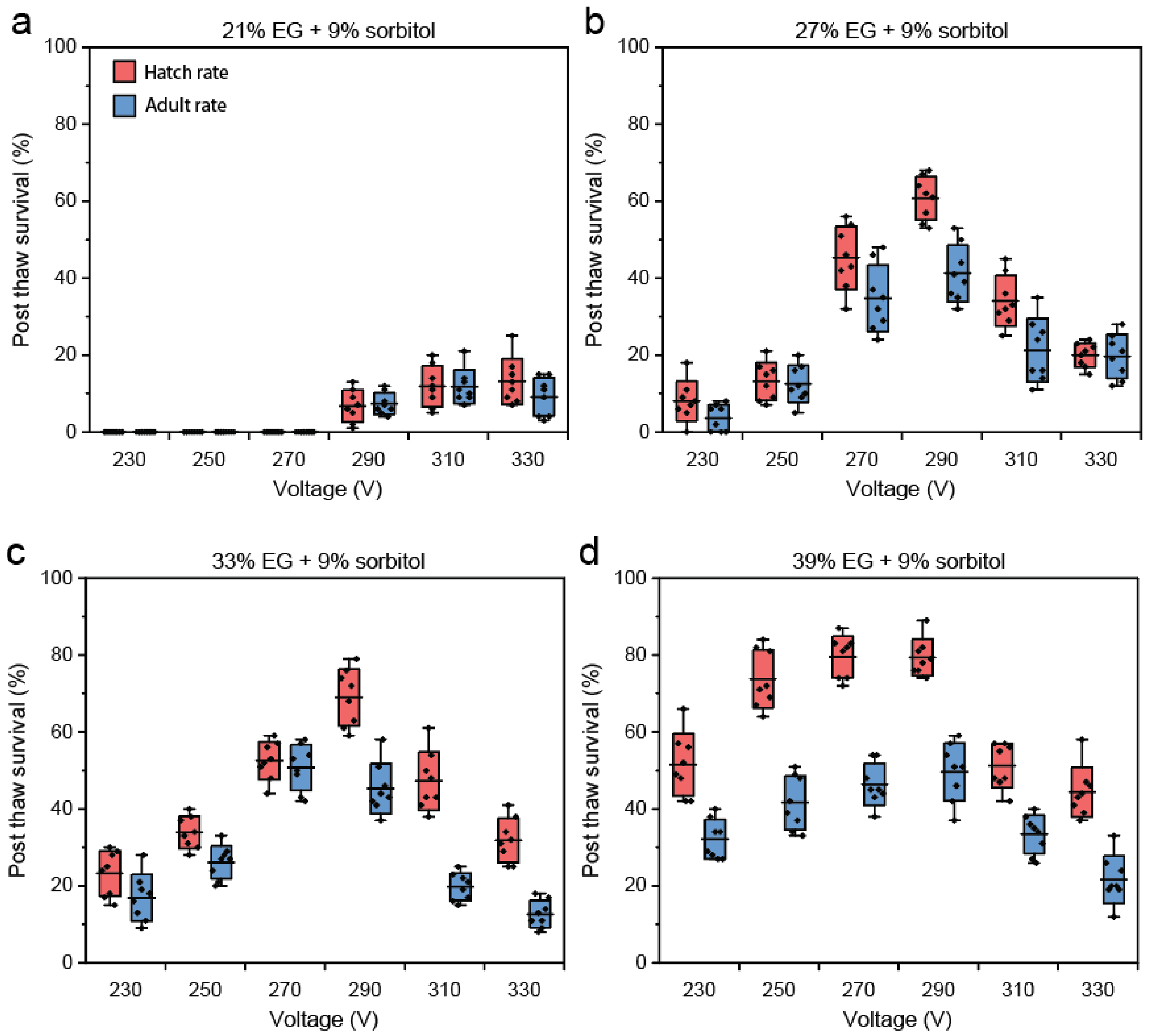

Supplementary Fig.5 Survival of *Drosophila* embryos after 1 ms joule heating using different voltages (230 – 330 V) and CPA concentrations. The tested CPAs include (a) 21% EG + 9% sorbitol, (b) 27% EG + 9% sorbitol, (c) 33% EG + 9% sorbitol and (d) 39% EG + 9% sorbitol. Hatch rate (red) represents the survival from embryos to larvae, adult rate (blue) represents the survival from larvae to adults. n=8 independent experiments. Bounds and horizontal line of box represent standard deviation and mean respectively; whiskers represent max and min.

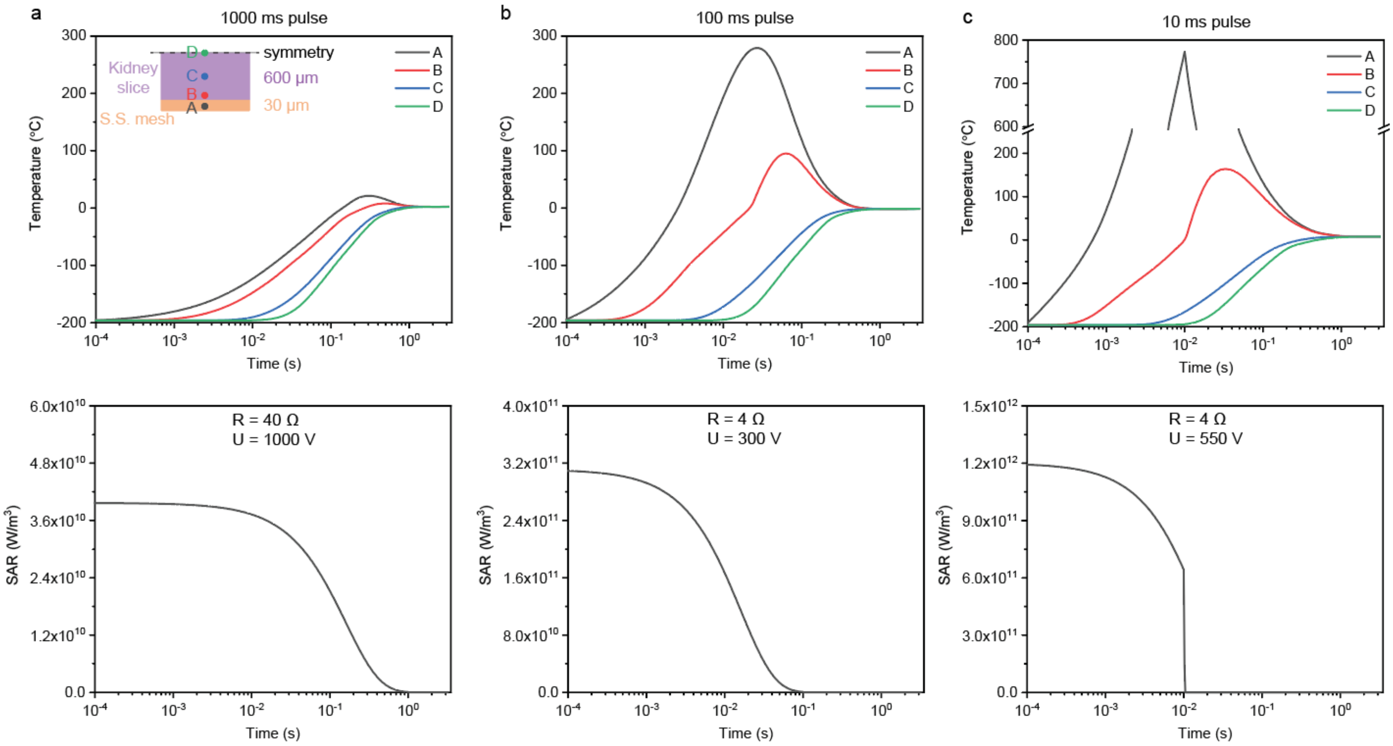

Supplementary Fig.6 The simulated temperature and specific absorption rate (SAR) profiles of the stainless steel (SS) mesh joule heating for kidney slices cryopreservation. The SAR profiles (lower panel) of 1000 ms (a), 100 ms (b), and 10 ms (c) voltage pulse were used to model the temperature profiles (upper panel). The resistance and voltage values were shown in the plot.

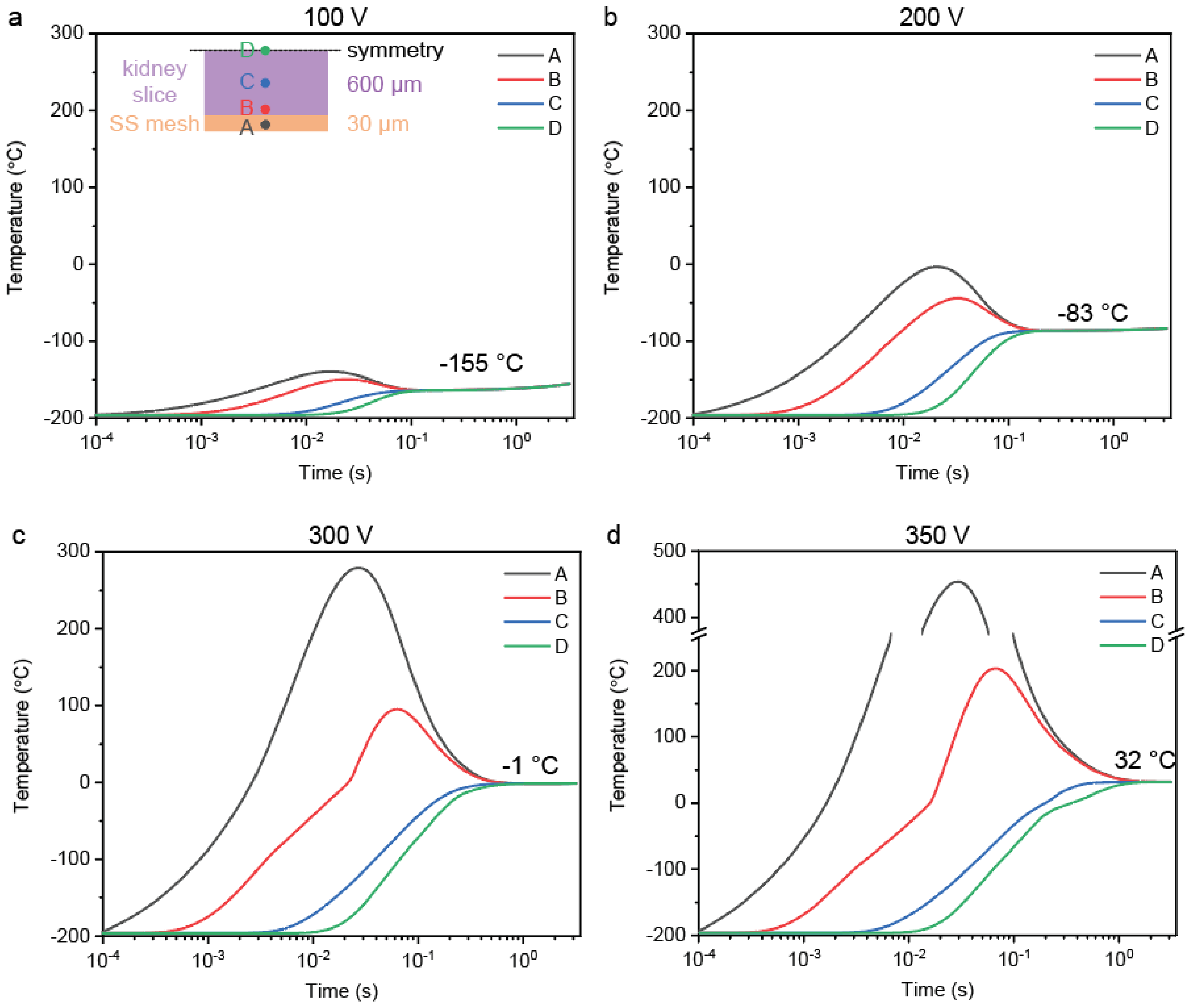

Supplementary Fig.7 The simulated temperature of the stainless steel (SS) mesh joule heating for 100 ms pulse rewarming of kidney slices using different voltages. Voltages including (a) 100 V, (b) 200 V, (c) 300 V, (d) 350 V were investigated. The final temperature after pulse heating is labelled in the plot.

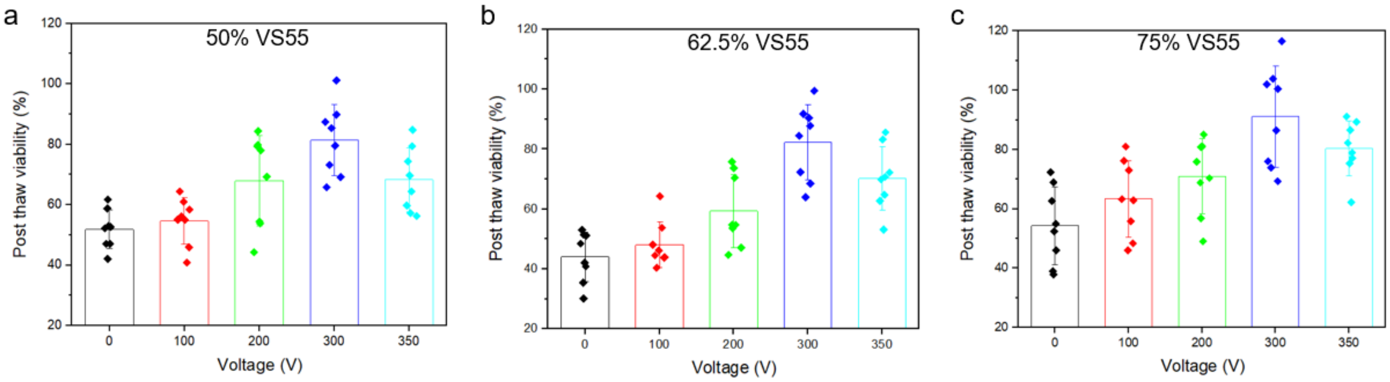

Supplementary Fig.8 Post thaw viability of the kidney slices by joule heating using different voltages and 100 ms pulse. The tested CPAs include (a) 50% VS55, (b) 62.5% VS55 and (c) 75% VS55. The viability was measured by alamarBlue assay and normalized by the readings prior to treatment. Data presented as mean ± s.d. n=8 independent samples.

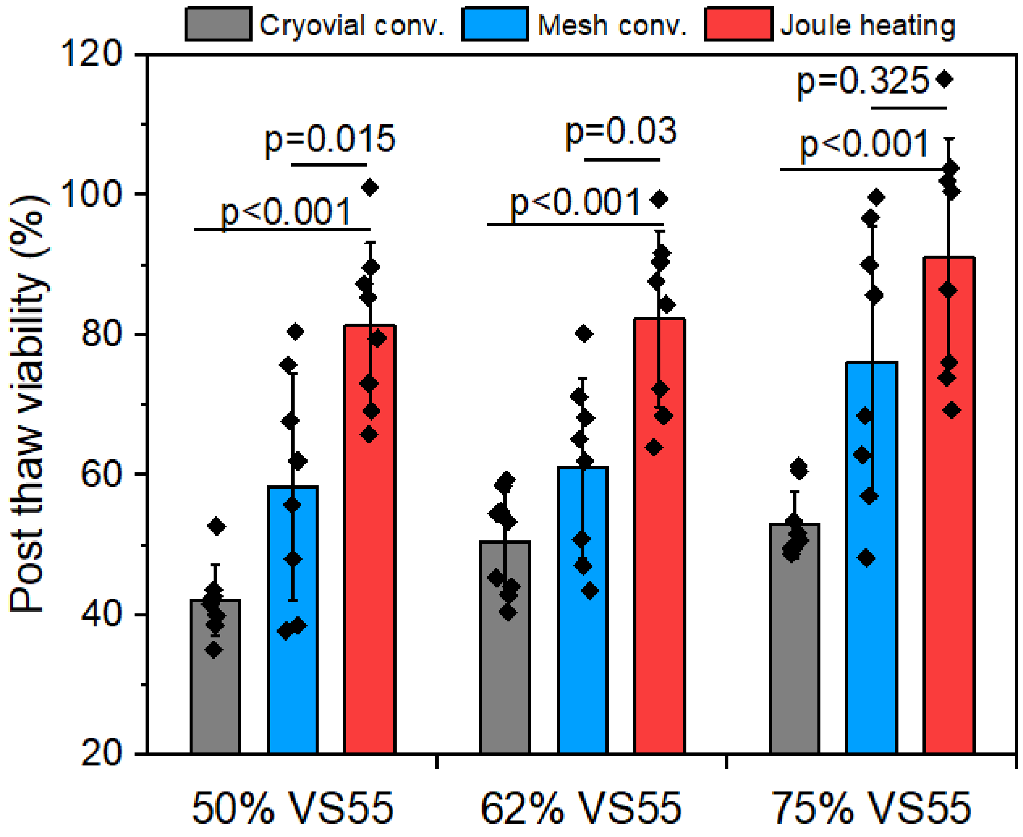

Supplementary Fig.9 Post thaw viability of kidney slices using different cryopreservation methods including cryovial convective warming, SS mesh convective warming and SS mesh joule heating. Different CPA concentrations including 50% VS55, 62% VS55 and 75% VS55 were tested (n=8 independent samples). Alamar blue was used to measure viability. Data are presented as mean values ± s.d. One-way ANOVA and Tukey's post hoc test were used.

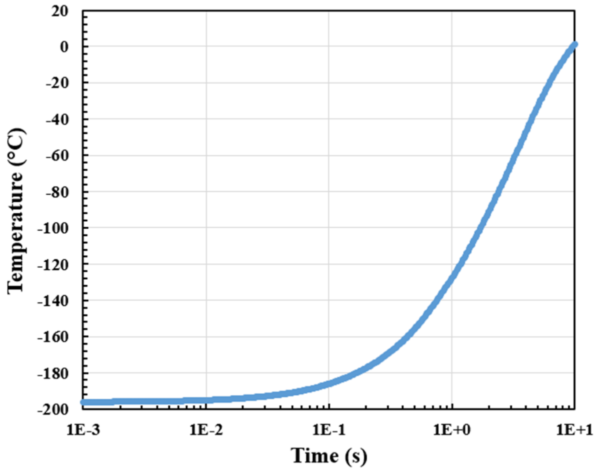

Supplementary Fig.10 Simulated temperature of adherent cells after being removed from liquid nitrogen due to natural convection in the air. No joule heating was applied.

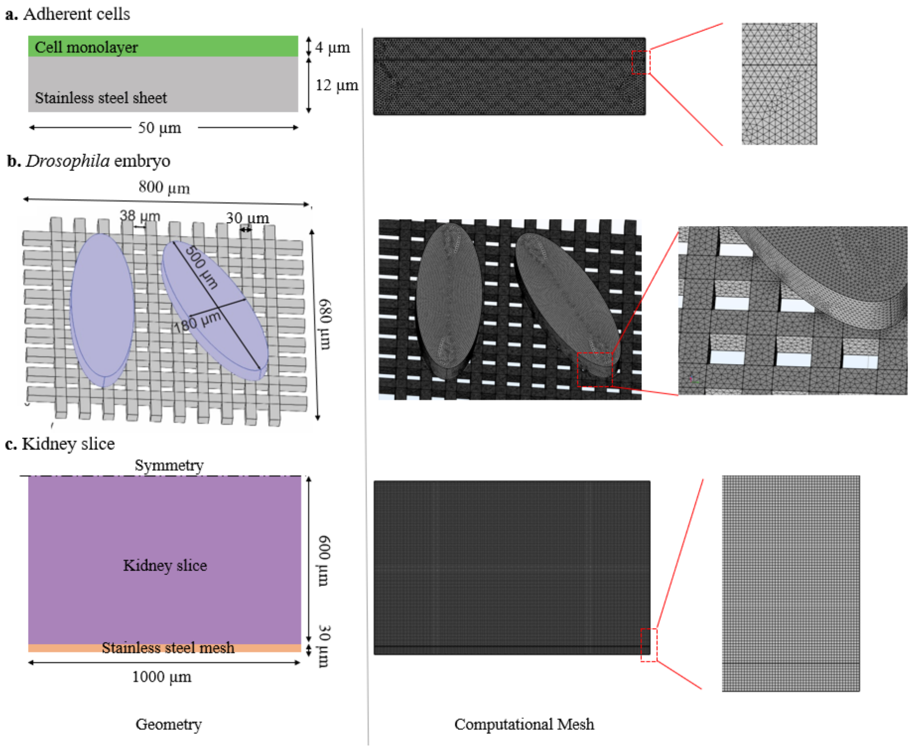

Supplementary Fig.11 The geometry and computational mesh used for heat transfer simulation of (a) adherent cells, (b) *Drosophila* embryos and (c) kidney slice in joule heating. The close-up views of the computational meshing are provided.
