# Supplementary material for: Rapid joule heating improves vitrification based cryopreservation
Source: Nat Commun. 2022 Oct 12;13:6017. doi: 10.1038/s41467-022-33546-9 (PMC9556611; doi:10.1038/s41467-022-33546-9)
Supplement: Supplementary file 3 — Description of Additional Supplementary Files [file 41467_2022_33546_MOESM3_ESM.pdf]

Description of Additional Supplementary Files

File name: Supplementary Movie 1.

Description: High speed video (3000 fps) of joule heating (290V, 1ms) of Drosophila embryos with 27% EG + 9% sorbitol as the CPA.

File name: Supplementary Movie 2.

Description: High speed video (3000 fps) of joule heating (290V, 1ms) of Drosophila embryos with 21% EG + 9% sorbitol as the CPA.
