# Supplementary material for: Rapid joule heating improves vitrification based cryopreservation
Source: Nat Commun. 2022 Oct 12;13:6017. doi: 10.1038/s41467-022-33546-9 (PMC9556611; doi:10.1038/s41467-022-33546-9)
Supplement: Supplementary file 6 — Reporting Summary [file 41467_2022_33546_MOESM6_ESM.pdf]

nature portfolio

Corresponding author(s): Li Zhan, John Bischof

Last updated by author(s): 2022-9-6

Reporting Summary

Nature Portfolio wishes to improve the reproducibility of the work that we publish. This form provides structure for consistency and transparency in reporting. For further information on Nature Portfolio policies, see our Editorial Policies and the Editorial Policy Checklist.

Statistics

For all statistical analyses, confirm that the following items are present in the figure legend, table legend, main text, or Methods section.

n/a	Confirmed	
☐	☒	The exact sample size (*n*) for each experimental group/condition, given as a discrete number and unit of measurement
☐	☒	A statement on whether measurements were taken from distinct samples or whether the same sample was measured repeatedly
☐	☒	The statistical test(s) used AND whether they are one- or two-sided<br>*Only common tests should be described solely by name; describe more complex techniques in the Methods section.*
☒	☐	A description of all covariates tested
☒	☐	A description of any assumptions or corrections, such as tests of normality and adjustment for multiple comparisons
☐	☒	A full description of the statistical parameters including central tendency (e.g. means) or other basic estimates (e.g. regression coefficient) AND variation (e.g. standard deviation) or associated estimates of uncertainty (e.g. confidence intervals)
☐	☒	For null hypothesis testing, the test statistic (e.g. *F*, *t*, *r*) with confidence intervals, effect sizes, degrees of freedom and *P* value noted<br>*Give P values as exact values whenever suitable.*
☒	☐	For Bayesian analysis, information on the choice of priors and Markov chain Monte Carlo settings
☒	☐	For hierarchical and complex designs, identification of the appropriate level for tests and full reporting of outcomes
☒	☐	Estimates of effect sizes (e.g. Cohen's *d*, Pearson's *r*), indicating how they were calculated

Our web collection on statistics for biologists contains articles on many of the points above.

Software and code

Policy information about availability of computer code

Data collection	COMSOL 5.5, BioTek's Gen5, EasyLogger 1.4 for DS1M12
Data analysis	Matlab 2019, ImageJ 1.52a, Graphpad 8

For manuscripts utilizing custom algorithms or software that are central to the research but not yet described in published literature, software must be made available to editors and reviewers. We strongly encourage code deposition in a community repository (e.g. GitHub). See the Nature Portfolio guidelines for submitting code & software for further information.

Data

Policy information about availability of data

All manuscripts must include a data availability statement. This statement should provide the following information, where applicable:

- Accession codes, unique identifiers, or web links for publicly available datasets
- A description of any restrictions on data availability
- For clinical datasets or third party data, please ensure that the statement adheres to our policy

All data are included in the main text and supplementary materials. Source data are provided in the paper.
The customized codes are freely available from Zenodo (https://doi.org/10.5281/zenodo.7054430).

Field-specific reporting

Please select the one below that is the best fit for your research. If you are not sure, read the appropriate sections before making your selection.

- [x] Life sciences
- [ ] Behavioural & social sciences
- [ ] Ecological, evolutionary & environmental sciences

For a reference copy of the document with all sections, see nature.com/documents/nr-reporting-summary-flat.pdf

Life sciences study design

All studies must disclose on these points even when the disclosure is negative.

Sample size	No sample size calculation was performed. We aimed to perform more than 3 replicates (i.e., independent experiments) for each experimental conditions, which is sufficient and commonly used to report viability results based on the literature.
Data exclusions	No data was excluded from the analysis
Replication	The number of replication in each experiment was represented by the number of data points in the presented figures. More than 3 replicates were performed in each experiment.
Randomization	Our sample were allocated randomly
Blinding	The samples were randomly selected and the outcome is determined by direct imaging and/or commercial readout device. Therefore the bias is minimized and the investigators were not blinded to different treatment groups.

Reporting for specific materials, systems and methods

We require information from authors about some types of materials, experimental systems and methods used in many studies. Here, indicate whether each material, system or method listed is relevant to your study. If you are not sure if a list item applies to your research, read the appropriate section before selecting a response.

Materials & experimental systems

n/a	Involved in the study	
x		Antibodies
	x	Eukaryotic cell lines
x		Palaeontology and archaeology
	x	Animals and other organisms
x		Human research participants
x		Clinical data
x		Dual use research of concern

Methods

n/a	Involved in the study	
x		ChIP-seq
x		Flow cytometry
x		MRI-based neuroimaging

Eukaryotic cell lines

Policy information about cell lines

Cell line source(s)	Human dermal fibroblasts were obtained from ATCC (PCS-201-012)
Authentication	The cell line has not been authenticated. We mainly focused on the viability after cryopreservation and the impact should be minimal.
Mycoplasma contamination	The cell lines were tested negative for mycoplasma.
Commonly misidentified lines (See ICLAC register)	none

Animals and other organisms

Policy information about studies involving animals; ARRIVE guidelines recommended for reporting animal research

Laboratory animals	Drosophila melanogaster were used. The strain information was provided in the Methods under the section of 'Drosophila embryos cryopreservation'. 1-15 days old male and female flies were used.<br>Male and female Sprague-Dawley rats (3-month old) were used in this study.
Wild animals	no wild animals were used in this study.

Field-collected samples	no field-collected samples were used in this study
Ethics oversight	All animal experiments were approved by the Institutional Animal Care and Use Committee (IACUC) at the University of Minnesota (IACUC Protocol: 1905-37029A)

Note that full information on the approval of the study protocol must also be provided in the manuscript.
