# Supplementary material for: Rapid joule heating improves vitrification based cryopreservation
Source: Nat Commun. 2022 Oct 12;13:6017. doi: 10.1038/s41467-022-33546-9 (PMC9556611; doi:10.1038/s41467-022-33546-9)
Supplement: Supplementary file 2 — Peer Review File [file 41467_2022_33546_MOESM2_ESM.pdf]

nature portfolio

Peer Review File

Rapid joule heating improves vitrification based cryopreservation

Open Access This file is licensed under a Creative Commons Attribution 4.0 International License, which permits use, sharing, adaptation, distribution and reproduction in any medium or format, as long as you give appropriate credit to the original author(s) and the source, provide a link to the Creative Commons license, and indicate if changes were made. In the cases where the authors are anonymous, such as is the case for the reports of anonymous peer reviewers, author attribution should be to 'Anonymous Referee' followed by a clear attribution to the source work. The images or other third party material in this file are included in the article's Creative Commons license, unless indicated otherwise in a credit line to the material. If material is not included in the article's Creative Commons license and your intended use is not permitted by statutory regulation or exceeds the permitted use, you will need to obtain permission directly from the copyright holder. To view a copy of this license, visit http://creativecommons.org/licenses/by/4.0/.

REVIEWER COMMENTS</B>

Reviewer #1 (Remarks to the Author):

I recognize that the submitted manuscript focuses on two aspects of 1) Development of heating device for thawing cryogenic biological matters up to sub-mm size in rapid thawing rate, 2) Sufficiently rapid thawing rate enhances quality of biological matters (cell, embryo, tissue). First aspect belongs to technology or engineering R&D rather than science, while the second aspect includes scientific findings or suggestion. Followings are the comments and the points that should be addressed, according to the guideline of this journal.

A) What are the noteworthy results?

１．Controllable Rapid heating Device
As a production process (or device) in industry, Joule heating contact plate (a conventional hot press Jour heater may be the most famous device) is already widely used as a device for rapid heating.
In this context, the principle or method of the devise itself is not new. However, the application of this method (or device) for rewarming biological samples at cryogenic temperature might be new point. The authors make effective use of the advantages of this method, which gives rapidly controllable large heat source to the contacted material in simple and low-cost ways, for rewarming the biological samples at cryogenic temperature that require rapid thawing to avoid devitrification or ice grain coarsening in the samples. In the manuscript, the results with analysis try to prove that the sample of up to sub mm thickness is uniformly rewarmed, because of this advantage.

２．The evaluation of the viability of biological samples after rewarming by the proposed method includes some scientific proofs or suggestions.
The experimental results shows that the rapid cooling followed by ultra-rapid warming largely enhance the viability of sample comparing with that followed by convection heating. There are many reports suggesting or proving that devitrification and ice grain coarsening have been proved to be the deleterious for biological cells or tissues (e.g., Biopreservation and Biobanking, Vol. 14, No. 6, Xiaoli Liu et.al.). In line of this, the enhancement of cellular viability by rapid rewarming includes no new scientific finding, but rather shows excellent case of realizing rapid thawing.
However, the results of this manuscript also suggest that even in case intracellular ice may exist (cells in low concentration VS), which believed to be lethal to cells, the ultra-rapid warming alleviates cellular damage (Fig.3e, the third paragraphs in Discussions). This may be the new finding that intracellular ice, in some cases, is not immediately causes cellular death.

B) Will the work be of significance to the field and related fields?

The proposed fast-controllable rapid heating device is simple, low cost and can be easily scale up the sample area. These advantages are the significant technology in the field of cryogenic bio-preservation.

C) How does it compare to the established literature?

The submitted manuscript is proposing the conventional technology that is excellently revised to new application field, rather than proposing new scientific finding.

D) Does the work support the conclusions and claims, or is additional evidence needed?

No additional evidence is required

E) Are there any flaws in the data analysis, interpretation and conclusions?

There are several minor typos and major points that should be addressed.

Minor typos:
1. Eq. (6): The right-hand side of this equation is temperature difference (unit K) caused by the single pulse. Thus, the left-hand side should be ΔT not ΔT/pw, which has a unit of K/sec. The same typo can be found in the main text just below the equation.
2. Results Joule heating for Adherent cell cryopreservation 2nd paragraph: (Fig.2b) in line 9 and 10 might be Fig.3b.
3. Results Joule heating for Adherent cell cryopreservation 3rd paragraph: (Fig.S2) in line 12 might be Fig.4c.
4. Fig.5 i: Arrows that point specific structure and anatomy mentioned in the main text are helpful for the potential readers.

Major questions:
1. Through the manuscript, the temperature in the biological sample after the pulse is defined by the heat transfer equation (Eq.(10)), which is especially important to discuss the homogeneity and the heating speed of the sample. Precisely, during warming, phase transition (melting) should happen in the biological sample by which the "effective" thermal diffusivity of the sample largely changes. Apart from the precise modelling, the "effective" thermal diffusivity of the sample used in each sample and its reason should be at least explained in the section Heat transfer modelling.

2. Temperature profile on the heating plate or mesh is also especially important to control the boundary condition of the sample. In this study, temperature was measured using thin film resistor thermometer (or thermocouple) with electrical insulated coat (e.g., 5th paragraph in section Characterization of joule heating in the RC discharge circuit), but less/no explanation about how it contact to the Joule heater. The thermal resistance between the thermometer and the heater might drastically change depending on their contact conditions, especially when a microscale water film, which cause phase change during freezing or thawing, is sandwiched by them. The condition of their contact should be explained for the reproducibility of experiments.

3. In the section Adherent cells cryopreservation, the pulse was applied "immediately after" the SS sheet was brought out of liquid nitrogen (2nd paragraph). How long does it take from sample bringing out to the pulse application? Since this system is very thin, the exposure to the room temperature might, within less than a second, cause the temperature rise at the surface of SS sheet where cells adhere. It may be better to mention the rough time scale of "immediately after".

4. In the section Kidney slices cryopreservation, a cryovial was used for convective warming in comparison to Joule heating method, in which a tissue slice was sandwiched by SS mesh strip. Why a cryovial was used, while for cryopreservation of other samples the samples were installed in the same SS plate or mesh as Joule heating method? To compare the effect of the heat transfer modes (Joule heater contact and convective heating), the system to be heated should be the same structure.

5. In the section Joule heating for Drosophila embryos cryopreservation, the pulse wide of 1msec was selected as the optimal pulse length. Looking at Fig.S3, the estimated temperature in the sample for the 1msec pulse has larger temperature gradient in the sample till 10msec after the pulse than that for the 10msec-pulse. Moreover, the warming speed at point A and B for the 1msec pulse seems slower above -50^oC, where devitrification temperature might be. This seems very strange for optimizing uniform-rapid rewarming. Explanation of physics or engineering that leads to select the 10msec-pulse is required.

6. In the Section of Physical limits of joule heating platform technique, and previous section, the parameter "heat diffusion time, td" appeals, definition of which is not clear (td=h²/α ?). Similarly, "heat diffusion length", the general definition of which is (α /tp)^{1/2}, is not clearly defined (= by equations.). Is this length

different from "Biosystem heat diffusion length"? Because of this, the original equations of the lines in Fig.6 a,b, especially in b are extremely difficult to derive. The explanation of these graphs in this section should need the background equations.

7. Physical meaning of the thermally induced injury model of Eq.(12) is not clear, and its reference (No. 50) in the references list is incomplete (no journal name or publishers name origin is unclear).

F) Do these(E))prohibit publication or require revision?
Yes

G) Is the methodology sound? Does the work meet the expected standards in your field?
Yes

H) Is there enough detail provided in the methods for the work to be reproduced?
Mostly, yes (please refer E) in detail)

I) The supplemented VTRs seem to be destructed.

Reviewer #2 (Remarks to the Author):

This is a very interesting and compelling study with cutting edge research on a highly challenging problem in the field of cryobiology. The authors report on a novel method to rewarm cryopreserved/vitrified samples, with the final aim to cryopreserve large biosystems and eventually whole organs. Even though this study does not report on organ preservation, it shows how a novel warming method can be used to rapidly rewarm cryopreserved biological samples of different sizes and geometries resulting in an improved cryopreservation outcome compared to standard convective rewarming. This method may hold promise for organ preservation.
Cryopreservation not only depends on the cooling conditions, but also on rewarming the sample at a high enough speed to avoid recrystallization/devitrification. The paper is well written with high quality illustrations. However, there are various issues that are not clear and need to be addressed.

Main points:
The choice of biosystems that were chosen here do not really have a biological or biomedical connection, i.e., attached fibroblast cells, drosophila embryos, and kidney tissue slices. Moreover, different vitrification solutions were used for the different systems, i.e., VS55 for tissue and attached cells, and EG/sorbitol for drosophila embryos. Some kind of motivation/rational should be given here.

The results section could be condensed a bit to increase readability and to highlight the main findings. For example, many numbers of the cooling rate at different pulse times are mentioned in the text while these numbers are also shown in the figures. I would recommend to shorten the results section and focus more on explaining the biophysical principles underlying joule heating.

Tissue viability was determined using an Alamar blue assay. This assay is based on functional esterases in cells. A fluorescent compound is formed in viable cells that can be visualized or measured using a fluorometer. It is mentioned that viability was inferred from fluorescence reading in the supernatant. It is not clear to me how this can be done since fluoresence comes from (intact) cells. This should be explained. In addition, pros and cons of using this assay for tissue viability should be discussed. Tissue viability is complex and requires many different functionality studies. There are limitations to only using a rather simple fluorescent Alamar blue assay to report tissue viability.

The heat simulation studies are only explained briefly in the MM section. The dimensions of the biosystem are not clear to me and only the height is mentioned. Did the authors use a mesh to describe the biological system, i.e., used a finite element method? Or where the simulations only done in one dimension?
Moreover, what were the material parameters that were used for the modeling? Parameters like heat capacity and CPA density are temperature dependent, and further change upon phase or state transitions. Have these parameters been derived from extrapolations? Do the material properties of the biosystem (cell /tissue material) also play a role? i.e, properties of the tissue extracellular matrix, and the lipid content in cells. How do these properties affect heat transfer?
More details are needed to explain how heat transfer simulations were done.

In the MM section, it is mentioned that: 'We used D = 6.5 × 10-11 m2/s, as reported in a previous study.21' What does this number reflect? There are multiple CPAs that were used for vitrification and each component is expected to permeate at a different rate. Moreover, the apparent diffusion coefficient is typically concentration dependent. This should be explained.

The authors compare their findings using joule heating with convective warming results. However, it would be more appropriate to compare the findings here with other warming methods.

RE: Response to reviews for NCOMMS-22-11474

Dear Reviewers,

We appreciate the time and thoughtful considerations that the Reviewers have given to our manuscript "Rapid joule heating improves vitrification based cryopreservation." We have incorporated those suggestions in a revised version for increased clarity and impact. We have provided a point-by-point response to all the comments (*in italics*) from the Reviewers below. We note the changes made to the manuscript and Supplementary Materials in response to the review in blue text.

Response to Reviewer #1:

I recognize that the submitted manuscript focuses on two aspects of 1) Development of heating device for thawing cryogenic biological matters up to sub-mm size in rapid thawing rate, 2) Sufficiently rapid thawing rate enhances quality of biological matters (cell, embryo, tissue). First aspect belongs to technology or engineering R&D rather than science, while the second aspect includes scientific findings or suggestion. Followings are the comments and the points that should be addressed, according to the guideline of this journal.

Response: We thank the reviewer for the summary of our work and positive comments. We agree with the reviewer that new scientific findings/suggestions can be enabled by careful engineering studies and novel technology development (or "R&D"). Besides offering scientific findings, our platform technology offers to improve a wide range of cryopreservation applications in a simple, scalable, and low-cost manner, and so should be of broad interest to the scientific community. In this revised form, we have strengthened our manuscript by providing additional details and explanations to clarify and address the questions raised by the reviewer as below:

A) What are the noteworthy results?

1. *Controllable Rapid heating Device*

As a production process (or device) in industry, Joule heating contact plate (a conventional hot press Jour heater may be the most famous device) is already widely used as a device for rapid heating.
In this context, the principle or method of the devise itself is not new. However, the application of this method (or device) for rewarming biological samples at cryogenic temperature might be new point. The authors make effective use of the advantages of this method, which gives rapidly controllable large heat source to the contacted material in simple and low-cost ways, for rewarming the biological samples at cryogenic temperature that require rapid thawing to avoid devitrification or ice grain coarsening in the samples. In the manuscript, the results with analysis try to prove that the sample of up to sub mm thickness is uniformly rewarmed, because of this advantage.

Response: We appreciate this comment. Although joule heating has been used for many other applications, our work highlighted the novel usage and carefully designed application of joule heating for rapid rewarming of cryogenically stored biological samples. The results demonstrate improved cryopreservation outcomes at low CPA concentrations in a simple, low-cost and scalable manner, which is a highly challenging problem in the field of cryobiology.

2. *The evaluation of the viability of biological samples after rewarming by the proposed method includes some scientific proofs or suggestions.*

The experimental results shows that the rapid cooling followed by ultra-rapid warming largely enhance the viability of sample comparing with that followed by convection heating. There are many reports suggesting or proving that devitrification and ice grain coarsening have been proved to be the deleterious for biological cells or tissues (e.g., Biopreservation and Biobanking, Vol. 14, No. 6, Xiaoli Liu et.al.). In line of this, the enhancement of

cellular viability by rapid rewarming includes no new scientific finding, but rather shows excellent case of realizing rapid thawing.
However, the results of this manuscript also suggest that even in case intracellular ice may exist (cells in low concentration VS), which believed to be lethal to cells, the ultra-rapid warming alleviates cellular damage (Fig.3e, the third paragraphs in Discussions). This may be the new finding that intracellular ice, in some cases, is not immediately causes cellular death.

Response: We thank the reviewer for this comment. Indeed, our results provide new evidence suggesting that ultra-rapid warming rates, provided by the joule heating approach, can rescue/alleviate damage due to intracellular ice formation during cooling at low concentration CPAs.

- We have added the following reference:

49. Liu, X., *et al.* Quantification of intracellular ice formation and recrystallization during freeze–thaw cycles and their relationship with the viability of pig iliac endothelium cells. *Biopreservation and biobanking* **14**, 511-519 (2016).

B) Will the work be of significance to the field and related fields?

The proposed fast-controllable rapid heating device is simple, low cost and can be easily scale up the sample area. These advantages are the significant technology in the field of cryogenic bio-preservation.

Response: We thank the reviewer for the positive comment.

C) How does it compare to the established literature?

The submitted manuscript is proposing the conventional technology that is excellently revised to new application field, rather than proposing new scientific finding.

Response: We thank the reviewer for this comment. Compared to the established literature in the cryogenic bio-preservation field, our joule heating approach showed the fastest and widest range of warming rates (Table 1) and demonstrated improved outcomes for cryopreserving biosystems using low concentration CPAs. Meanwhile, as the reviewer mentioned in the previous comment, the ultra-rapid joule heating technique allows us to directly demonstrate a new finding. At both cellular (Figure 3e) and organismal (Figure 4f, 4j, Supplementary movies 1-2) level, ultra-rapid joule heating can "rescue" biosystems that develop some ice during cooling by outracing the lethal ice crystal growth and cellular damage during warming. These findings advance the scientific field's understanding of possible rates for rewarming and mechanisms of damage and rescue from intracellular ice formation.

D) Does the work support the conclusions and claims, or is additional evidence needed?

No additional evidence is required

Response: We thank the reviewer for this comment.

E) Are there any flaws in the data analysis, interpretation and conclusions?

There are several minor typos and major points that should be addressed.

Minor typos:

1. *Eq. (6): The right-hand side of this equation is temperature difference (unit K) caused by the single pulse. Thus, the left-hand side should be ΔT not ΔT/pw, which has a unit of K/sec. The same typo can be found in the main text just below the equation.*

Response: We thank the reviewer for pointing this out. We have fixed the typo in Eq. (6). We also noted a typo in Eq. (3).

- We have made the following corrections in the main text and figure:

Page 16 of the manuscript:

$$Q = \frac{CR^2 I_0{}^2}{2}\left(1 - e^{-\frac{2pw}{RC}}\right) = mC_p\Delta T \quad (3)$$

$$\Delta T = \frac{CI_0{}^2}{2}\left(1 - e^{-\frac{2pw}{RC}}\right)\frac{R^2}{mC_p} \propto \frac{R^2}{mC_p} = \frac{L}{A^3}\frac{1}{\sigma^2 \rho C_p} \quad (6)$$

Page 5 of the manuscript:

Under the same electrical current, material dimensions and pulse width, we found that warming rate is inversely correlated to three material properties including density (ρ), electrical conductivity (σ), and heat capacity (Cp). As shown in Fig. 2d, among the commonly used metal materials, SS provides the highest warming rate (i.e., lowest value of $\rho*\sigma^2*Cp$).

Figure 2d:

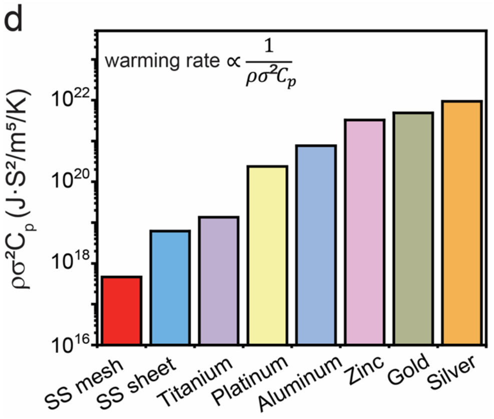

2. *Results Joule heating for Adherent cell cryopreservation 2nd paragraph: (Fig.2b) in line 9 and 10 might be Fig.3b.*

Response: We thank the reviewer for the careful read of our manuscript. We have corrected the typos in the revised manuscript.

3. *Results Joule heating for Adherent cell cryopreservation 3rd paragraph: (Fig.S2) in line 12 might be Fig.4c.*

Response: We apologize for the confusion. We have corrected the figure number to "Fig. S3" on page 9 of the revised manuscript.

4. *Fig.5 i: Arrows that point specific structure and anatomy mentioned in the main text are helpful for the potential readers.*

Response: We thank the reviewer for this suggestion. We have added arrows in Fig. 5i and updated the main text for clarification.

- Updated Fig. 5i:

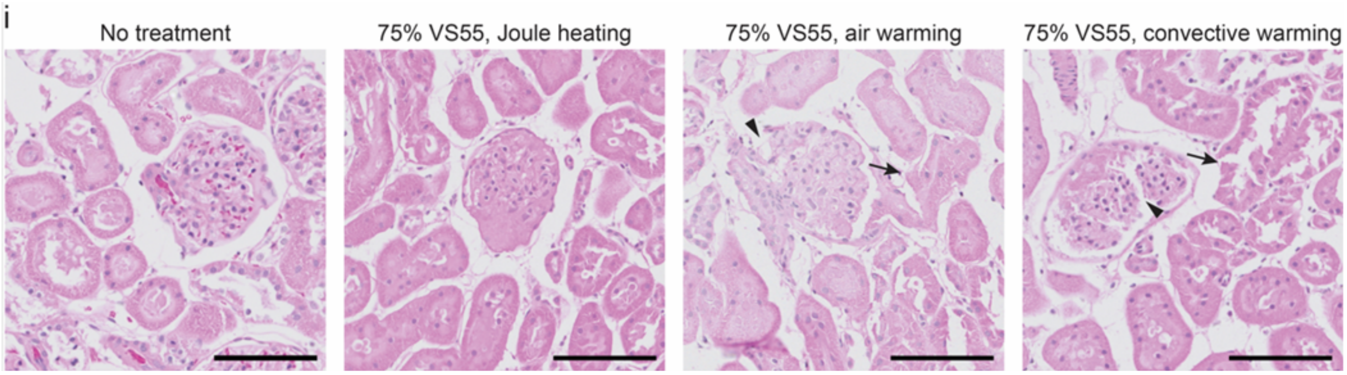

Page 34 of the manuscript:

i. Histology (H & E staining) of kidney slices. Arrowhead showed disrupted glomeruli, and arrow indicated damaged proximal tubules.

Major questions:

1. *Through the manuscript, the temperature in the biological sample after the pulse is defined by the heat transfer equation (Eq.(10)), which is especially important to discuss the homogeneity and the heating speed of the sample. Precisely, during warming, phase transition (melting) should happen in the biological sample by which the "effective" thermal diffusivity of the sample largely changes. Apart from the precise modeling, the "effective" thermal diffusivity of the sample used in each sample and its reason should be at least explained in the section Heat transfer modelling.*

Response: We thank the reviewer for this comment. For the temperature profile simulation of biological systems (Eq. 10), we applied temperature-dependent density (ρ), heat capacity (C_p) and thermal conductivity (k), which allow us to capture the change of thermal diffusivity ($\alpha = k/(\rho C_p)$) during the phase transition process.
Due to the lack of experimental data on the specific biosystems (i.e., cell, embryo, tissue) tested in current study, we selected the thermal properties from previously published results of relevant samples in the literature. For heat capacity, CPA-loaded biological samples have relatively similar values with CPA solutions[1-4]. We used the temperature dependent, experimentally measured (i.e., by DSC) heat capacity of VS55[1] as the approximation for simplicity. For thermal conductivity, we used the reported values for 6 M glycerol-laden porcine liver[4] as the estimation for the biosystems tested in this study. Temperature-dependent density of VS55[2] was used for all biosystems in the simulation as the estimation. For simplicity, we used the same material properties for all biosystems (cell, embryo and tissue).

- To clarify this, we have added the following sentences to page 19 of the manuscript:

For various biosystems (adherent cells, *Drosophila* embryos, kidney slices), in the absence of experimental data on the material properties, we used the thermal properties of relevant samples as previously reported in the literature. Specifically, temperature dependent density of VS55[50] was used for all biosystems. The reported temperature dependent thermal conductivity of 6 M glycerol-laden porcine liver[42] was used as the estimation for all biosystems. For heat capacity, similar thermal properties have been reported between CPA solutions and CPA-loaded biological samples[21,25,50], we used the experimentally measured values for VS55[25] as the approximation for

all biosystems. More detailed characterization of the thermal properties for CPA-laden biosystems (cells, embryos and tissues) is needed in the field, but our simulation should provide an initial approximation of the heat transfer process.

2. *Temperature profile on the heating plate or mesh is also especially important to control the boundary condition of the sample. In this study, temperature was measured using thin film resistor thermometer (or thermocouple) with electrical insulated coat (e.g., 5th paragraph in section Characterization of joule heating in the RC discharge circuit), but less/no explanation about how it contact to the Joule heater. The thermal resistance between the thermometer and the heater might drastically change depending on their contact conditions, especially when a microscale water film, which cause phase change during freezing or thawing, is sandwiched by them. The condition of their contact should be explained for the reproducibility of experiments.*

Response: We thank the reviewer for this important comment. To measure the temperature of the commercial thin film resistor which has an electrically insulated coating surface, we attached a thermocouple to the thin film resistor using scotch tape such that the thermocouple junction (50 µm in size) is in close contact with the surface of the heater (i.e., thin film resistor). No aqueous solution was involved in this measurement, and we observed reproducible temperature measurement during cooling and joule heating of the thin film resistor (Fig. 2h, 2j). During cryopreservation of different biological systems, we did not report direct temperature measurement of the heater (i.e., stainless steel sheet and/or mesh), due to the lack of electrical insulation coating on the stainless-steel materials we used.

- We have added the following details to page 15 of the manuscript to clarify this:

An ultrafine gauge type T thermocouple (COCO-002, OMEGA) was attached to the surface of a resistor using scotch tape to ensure close contact. No aqueous solution was involved during this measurement.

3. *In the section Adherent cells cryopreservation, the pulse was applied "immediately after" the SS sheet was brought out of liquid nitrogen (2nd paragraph). How long does it take from sample bringing out to the pulse application? Since this system is very thin, the exposure to the room temperature might, within less than a second, cause the temperature rise at the surface of SS sheet where cells adhere. It may be better to mention the rough time scale of "immediately after".*

Response: We thank the reviewer for this thoughtful comment. It takes 0.25 ~ 0.4s (measured by a stopwatch) to bring out the SS sheet to the pulse application. We conducted the heat transfer modeling in COMSOL to check the temperature increase of the cells due to convective warming assuming convective heat transfer coefficient of 10 $W/m^2/K$ in air. As shown in the figure below (Fig. S9), after 0.25 ~ 0.4 s, the temperature of cells increased to -173 ~ - 161 °C, which is still below the glass transition temperature (i.e., usually higher than -140 °C), indicating minimal risk of devitrification during this process.

- We have added the following sentences to page 17 of the manuscript:

Using a stopwatch, we measured that it took 0.25 ~ 0.4 s to bring out the SS sheet from liquid nitrogen until the application of joule heating pulse. Assuming a convective heat transfer coefficient of 10 $W/m^2/K$ in air, heat transfer modeling suggested that the temperature of cells increased to -173 ~ -161 °C (Fig. S9), still below the glass transition temperature.

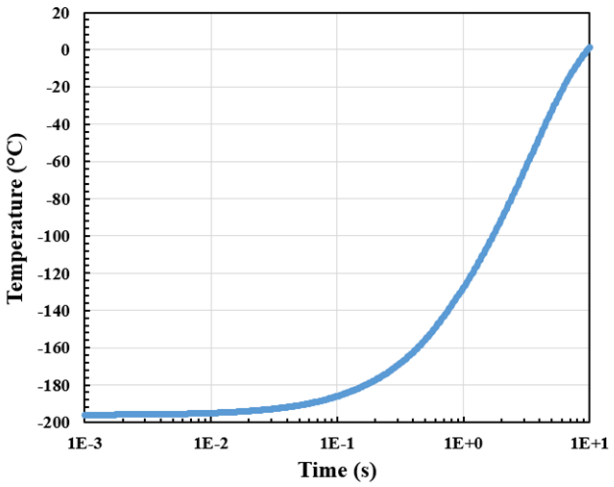

Fig. S9. Simulated temperature of adherent cells after being removed from liquid nitrogen due to natural convection in the air. No joule heating was applied.

4. *In the section Kidney slices cryopreservation, a cryovial was used for convective warming in comparison to Joule heating method, in which a tissue slice was sandwiched by SS mesh strip. Why a cryovial was used, while for cryopreservation of other samples the samples were installed in the same SS plate or mesh as Joule heating method? To compare the effect of the heat transfer modes (Joule heater contact and convective heating), the system to be heated should be the same structure.*

Response: We thank the reviewer for this comment. We selected convective warming of a cryovial because it is the standard method used in the literature for tissue slice cryopreservation[5-9]. However, we agree with the reviewer that an experiment where slices were warmed between convectively warmed SS mesh and compared to slices warmed between joule heated SS mesh would be instructive and we have therefore added it. We now present Fig. S11 below which shows the post-thaw viabilities (measured by alamar blue) for 50% VS55, 62.5% VS55, and 75% VS55 were 54.9 ± 17%, 61 ± 12%, and 76.1 ± 18.2% using SS mesh convective warming which are significantly lower than the survival rates of SS mesh joule heating.

- To address this, we have added the following sentences to the manuscript:

Page 11 of the manuscript:

For direct comparison of SS mesh convective warming and joule heating, the kidney slices were sandwiched between the SS mesh, plunged into liquid nitrogen and then convectively rewarmed. The post-thaw viabilities for 50% VS55, 62.5% VS55, and 75% VS55 were 42.1 ± 4.8%, 50.3 ± 6.8%, and 52.9 ± 4.5% using cryovial convective warming; 54.9 ± 17%, 61 ± 12%, and 76.1 ± 18.2% using SS mesh convective warming (Fig. S11); and 81.4 ± 11%, 82.3 ± 11.8%, and 91.1 ± 15.9% using SS mesh joule heating, respectively (Fig. 5h).

Figure S11:

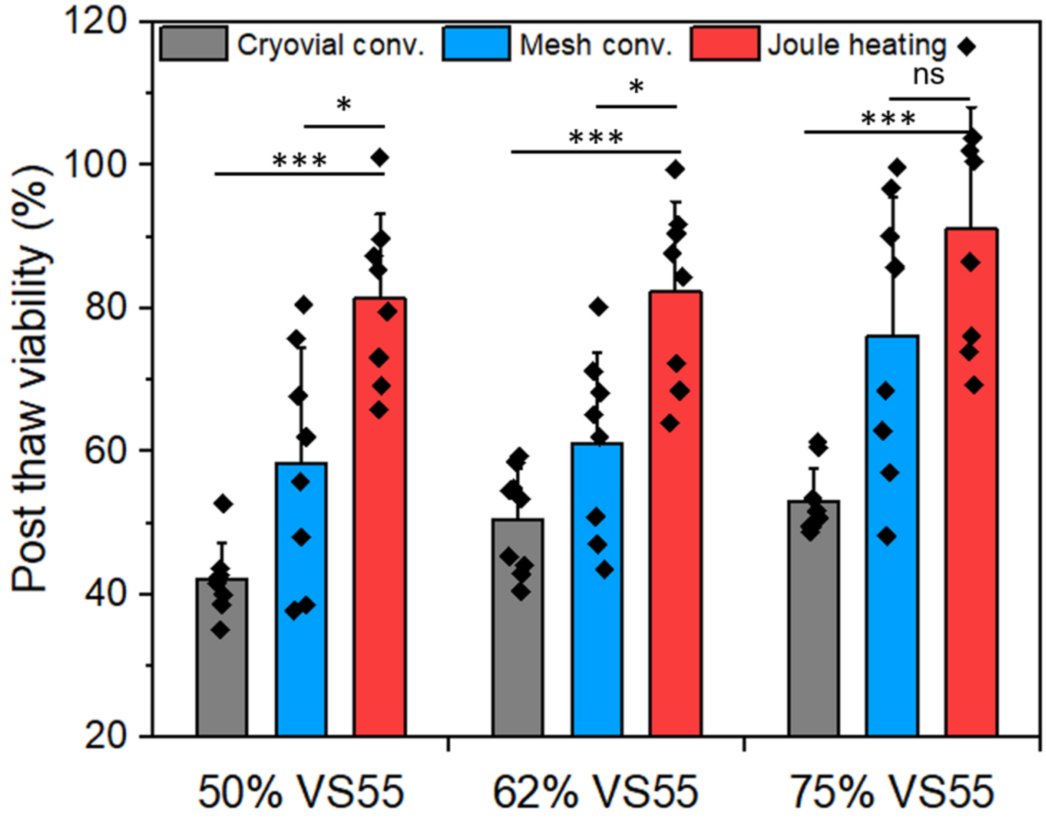

Figure S11. Post thaw viability of kidney slices using different cryopreservation methods including cryovial convective warming, SS mesh convective warming and SS mesh joule heating. Different CPA concentrations including 50% VS55, 62% VS55 and 75% VS55 were tested (n=8). Alamar blue was used to measure viability. One-way ANOVA and Tukey's post hoc test were used: *ns, p>0.05, * p<0.05, ** p<0.01, *** p<0.001.*

5. *In the section Joule heating for Drosophila embryos cryopreservation, the pulse wide of 1msec was selected as the optimal pulse length. Looking at Fig.S3, the estimated temperature in the sample for the 1msec pulse has larger temperature gradient in the sample till 10msec after the pulse than that for the 10msec-pulse. Moreover, the warming speed at point A and B for the 1msec pulse seems slower above -50ºC, where devitrification temperature might be. This seems very strange for optimizing uniform-rapid rewarming. Explanation of physics or engineering that leads to select the 10msec-pulse is required.*

Response: We thank the reviewer for noting the pulse width selection for rewarming *Drosophila* embryos. Since the embryos were rewarmed from the boundaries, there is an intrinsic trade-off between rapid and uniform rewarming. For instance, rewarming failures can occur due to slow warming rate (i.e., lethal ice formation from devitrification), or thermal stress induced injuries (i.e., cracking caused by non-uniform rewarming), or a combination of both. Our strategy to avoid rewarming failures is to prioritize the rapid warming rate while ensuring "sufficiently" uniform rewarming (i.e., no visible cracking of the embryos or sharp decrease of viability). Therefore, compared with 10 msec pulse, we selected 1 msec pulse to achieve faster warming rate of the embryos (warming rates comparison shown in Fig. 4c). Indeed, we did not observe visible cracking or leakage of embryos post rewarming, and the viability was high when no visible ice formed during rewarming (Fig. 4g, 4k, 39% EG + 9% sorbitol as the CPA), suggesting the temperature gradient during rewarming by 1msec pulse joule heating (Fig. S3b) is tolerable in the *Drosophila* embryos.
Regarding the comments about warming speed for temperature above -50 ºC, the calculated warming rates using temperature zone from -50 ºC to -10 ºC for point A and B are 5.9×10^5 and 6.6×10^5 °C/min for 1 ms pulse; 3.8 ×

10^5, 4.2×10^5 °C/min for 10 ms pulse. Thus, 1 ms pulse provided faster warming rates than 10 msec for temperature above -50 ºC.

- We have added the following sentences to page 9 of the manuscript for clarification:

We selected a 1 ms pulse (comparable with t_d = 1.3 ms) to prioritize fast warming rates while avoiding large temperature gradients inside the embryo, compared with 0.1 ms and 10 ms pulses (Fig. 4c, S3).

Page 10 of the manuscript:

For 39% EG group, no visible lethal ice was recorded during rewarming by 1 ms pulse joule heating (Fig. 4g) and high viability was observed (Fig. 4k), suggesting the temperature gradient during rewarming (Fig. S3b) was tolerable in the *Drosophila* embryos.

6. *In the Section of Physical limits of joule heating platform technique, and previous section, the parameter "heat diffusion time, td" appeals, definition of which is not clear ($t_d=h^2/\alpha$?). Similarly, "heat diffusion length", the general definition of which is $(\alpha /tp)^{1/2}$, is not clearly defined (= by equations.). Is this length different from "Biosystem heat diffusion length"? Because of this, the original equations of the lines in Fig.6 a,b, especially in b are extremely difficult to derive. The explanation of these graphs in this section should need the background equations.*

Response: We thank the reviewer for this important suggestion. For each biosystem, the concept of diffusion length (h) can be found in heat transfer texts and literature as $h^2 = 2^* \alpha *t$,[10,11] as the scaling the order of magnitude of how far heat can diffuse within certain time. We are therefore only rewriting this well -established scaling equation to emphasize the heat diffusion time (t_d) as $t_d = h^2/2\alpha$, where h is heat diffusion length of the biosystem, and α is thermal diffusivity of the biosystem. Here, the heat diffusion length (h) is defined as the distance within the biosystem across which heat needs to diffuse from the boundary heater (i.e., stainless steel sheet or mesh in this study) to rewarm the biosystem. Specifically, the heat diffusion lengths for adherent cells and *Drosophila* embryos is equal to their thicknesses. For kidney slices, the heat diffusion length is half the thickness as they are being rewarmed from both sides (Fig. 5a). Overall, the full characterization of heat diffusion depends on the configuration of the biosystems and boundary heater, which would require solution of the full heat equation. We wished to avoid this in order to emphasize the diffusion scaling law of the system.
This heat diffusion length is the same as "Biosystem heat diffusion length" shown in Fig. 6. The equation to generate Fig. 6a is $t_d = h^2/2\alpha$. The equation to generate Fig. 6b is biosystem warming rate = 200ºC / t_p, where 200 ºC is the temperature difference from liquid nitrogen (-196 ºC) to 4 ºC.

- To clarify this, we have replaced "heat diffusion length" with "biosystem heat diffusion length" throughout the manuscript.

We have added the following sentences to page 7 of the manuscript:

In this study, we define biosystem heat diffusion length (h) as the distance within the biosystem across which heat needs to diffuse from the boundary heater (i.e., stainless steel sheet or mesh in this study) to rewarm the biosystem.

and the following on Page 12 of the manuscript:

The estimated overall warming rates can be calculated as *200 ºC /* t_p, where 200 ºC is the temperature difference from liquid nitrogen (-196 ºC) to 4 ºC.

7. *Physical meaning of the thermally induced injury model of Eq.(12) is not clear, and its reference (No. 50) in the references list is incomplete (no journal name or publishers name origin is unclear).*

Response: We thank the reviewer for this comment. The thermally induced injury model (Eq.12) has been widely used to evaluate the tissue damage (i.e., drop in viability) after exposure to high temperature in various scenarios such as hyperthermia for cancer treatment. In Fig. 5c, our numerical simulation suggested that part of the kidney slices (i.e., point B) may experience high temperature (40 to approximately 94 °C) for a short period of time (30 ms to 170 ms) during rewarming. We sought to apply the thermally induced injury model to estimate the resultant damage.
We have modified the reference to include the journal name and publishers name in the correct format.

- We have changed the following to page 11 of the manuscript:

We used the Arrhenius model to estimate the potential for thermally induced injury and found negligible impact on viability (i.e., 3.2% decrease in viability, details in Methods).

F) Do these(E))prohibit publication or require revision?
Yes

Response: We thank the reviewer for your valuable input and have integrated the reviewer's suggestions in the revised manuscript which we now believe is ready for publication.

G) Is the methodology sound? Does the work meet the expected standards in your field?
Yes

Response: We thank the reviewer for the positive comment.

H) Is there enough detail provided in the methods for the work to be reproduced?
Mostly, yes (please refer E) in detail)

Response: We have modified the manuscript following the reviewer's comments to improve clarity.

I) The supplemented VTRs seem to be destructed.

Response: We have re-uploaded the supplementary movie files.

<u>Response to Reviewer #2:</u>

1. *This is a very interesting and compelling study with cutting edge research on a highly challenging problem in the field of cryobiology. The authors report on a novel method to rewarm cryopreserved/vitrified samples, with the final aim to cryopreserve large biosystems and eventually whole organs. Even though this study does not report on organ preservation, it shows how a novel warming method can be used to rapidly rewarm cryopreserved biological samples of different sizes and geometries resulting in an improved cryopreservation outcome compared to standard convective rewarming. This method may hold promise for organ preservation. Cryopreservation not only depends on the cooling conditions, but also on rewarming the sample at a high enough speed to avoid recrystallization/devitrification. The paper is well written with high quality illustrations. However, there are various issues that are not clear and need to be addressed.*

Response: We thank the reviewer for the enthusiastic comments and encouragement.

2. *Main points:*
   The choice of biosystems that were chosen here do not really have a biological or biomedical connection, i.e., attached fibroblast cells, drosophila embryos, and kidney tissue slices. Moreover, different vitrification solutions were used for the different systems, i.e., VS55 for tissue and attached cells, and EG/sorbitol for drosophila embryos. Some kind of motivation/rational should be given here.

Response: We thank the reviewer for this suggestion. The selected biosystems were chosen to represent different length scales of biological samples to be cryopreserved (i.e. model systems). We aim to show that joule heating, as a platform technology, can be tuned accordingly to achieve warming rates in a wide range to rewarm biosystems with different biosystem heat diffusion lengths (i.e., thicknesses) including attached fibroblast cells (~ 4 µm), *Drosophila* embryos (~ 50 µm) and kidney slices (~ 1.2 mm).
Different CPA recipes were used based on our previous work and literature. Appropriate choice of CPA is highly dependent on the biological system chosen. Specifically, VS55 has been used extensively for cryopreservation of adherent cells, tissues and organs in the literature[3,12-14]. However, for *Drosophila* embryos, our previous work showed that DMSO (i.e., one of the major components in VS55) has higher toxicity than EG, and demonstrated successful cryopreservation with EG/sorbitol[15].

- We have modified the text on page 5 of the manuscript:

To investigate whether joule heating can serve as a platform technology to achieve rapid warming rates in a wide range to rewarm biosystems at different scales (i.e., thicknesses), we selected the adherent fibroblast cells (~ 4 µm), *Drosophila* embryos (~ 50 µm), and rat kidney slices (~ 1.2 mm) as the model systems (Fig. 1b). VS55 has been extensively used as the CPA for various biosystems[20-22] and was used to cryopreserve adherent cells and kidney slices in this study. For *Drosophila* embryos, EG/sorbitol showed successful cryopreservation in our previous work and was therefore used here[17].

3. *The results section could be condensed a bit to increase readability and to highlight the main findings. For example, many numbers of the cooling rate at different pulse times are mentioned in the text while these numbers are also shown in the figures. I would recommend to shorten the results section and focus more on explaining the biophysical principles underlying joule heating.*

Response: We thank the reviewer for this suggestion. We have now removed the sections listing the warming rates from main text.

4. *Tissue viability was determined using an Alamar blue assay. This assay is based on functional esterases in cells. A fluorescent compound is formed in viable cells that can be visualized or measured using a fluorometer. It is mentioned that viability was inferred from fluorescence reading in the supernatant. It is not clear to me how this can be done since fluoresence comes from (intact) cells. This should be explained. In addition, pros and cons of using this assay for tissue viability should be discussed. Tissue viability is complex and requires many different functionality studies. There are limitations to only using a rather simple fluorescent Alamar blue assay to report tissue viability.*

Response: We thank the reviewer for this comment. Alamar blue has been regularly used as an oxidation-reduction indicator for viability evaluation. Following incubation with the cells, alamar blue (i.e., resazurin) is reduced into resorufin, which is fluorescent and extracted from the cells into the medium. Therefore, the fluorescence of the supernatant can be used to represent the viability[16]. Using supernatant medium for alamar blue assay has been reported in numerous studies to assess various biological samples including tissue slices[17-19].
For tissue viability assessment, alamar blue has the advantages of low toxicity, high sensitivity, simplicity-of-use and extensive usage in the literature. The disadvantages include the semiquantitative nature of the assay and dependence on the metabolic pathway which can be affected by cellular status. We recognized that tissue viability

may not be fully represented by the alamar blue assay, and therefore also performed complementary histological examination of H&E stained sections of kidney slices. The histology observations corroborated the viability measurement by alamar blue. Our current work provides a broad proof-of-concept demonstration of a joule heating platform and related scientific findings. The optimization of cryopreservation protocols for each biological system will require more extensive evaluation on viability and function.

- We added the following text to page 19 of the manuscript:

For the assessment of tissue viability, alamar blue (i.e., resazurin) is reduced to a fluorescent derivative, resorufin, following incubation with cells/ tissues. Resorufin fluorescence is extracted from the cells into the medium and the fluorescence of the supernatant can be monitored as a proxy for viability. Alamar blue has the advantages of low toxicity, high sensitivity, simplicity-of-use and extensive usage in the literature. The disadvantages include the semiquantitative nature of the assay and dependence on the metabolic pathway which can be affected by cellular status.

Page 11/12 of the manuscript:

Overall, the alamar blue viability measurement was corroborated by the histological results, demonstrating that joule heating shows great promise in improving cryopreservation of millimeter-scale thickness tissues using low CPA concentrations. More extensive examination of tissue functionality will be important in future technology development.

5. *The heat simulation studies are only explained briefly in the MM section. The dimensions of the biosystem are not clear to me and only the height is mentioned. Did the authors use a mesh to describe the biological system, i.e., used a finite element method? Or where the simulations only done in one dimension?*

Response: We thank the reviewer for this comment. For heat transfer simulation, we selected the geometry to capture the key dimensions affecting the heat transfer and minimize the cost of computational resources. The detailed explanations for different biosystems (adherent fibroblast cells, *Drosophila* embryos, kidney slices) are provided as below:

a. Adherent fibroblast cells. As the cells formed a monolayer covering the surface of stainless-steel sheet (Fig. 3c, 3f) and the heat generation (SAR in Eq. 9) is homogenous, we sampled a 2D area (50 μm width, shown below) from the cross-section perpendicular to the length of the stainless steel sheet for the simulation.
b. *Drosophila* embryos. After CPA dehydration, the thickness of the embryo was reduced to 50 μm. The size of a single drosophila embryo (500 * 180 * 50 μm, Fig. 4b) is on the same order of magnitude with the stainless-steel mesh (wire diameter 30 μm, aperture size 38 μm). Therefore, we used a 3D model for the simulation as shown below (Fig. S10).
c. Kidney slices. The kidney slices (1.2 mm thickness, 6 mm diameter) were sandwiched and rewarmed from both sides using stainless steel mesh (Fig. 5a). We used a 2D area with dimensions shown below in the modeling (Fig. S10).

In all biosystems, finite element methods (i.e., with computational meshing) were used. The computational meshing used for different biosystems are shown in Fig. S10.

- We have added the following sentences to page 19/20 of the manuscript:

For different biosystems, we selected the geometry to capture the key dimensions affecting the heat transfer and minimize the cost of computational resources. For adherent cells, a 2D area (50 μm width, capturing the thickness of the cell monolayer and S.S. sheet, Fig. S10) from the cross-section perpendicular to the length of S.S. sheet was used. After CPA dehydration, the thickness of the *Drosophila* embryo was reduced to 50 μm. A 3D geometry (Fig. S10) was used as the sizes of a *Drosophila* embryo (500 * 180 * 50 μm) and SS mesh (wire diameter 30 μm,

aperture size 38 µm) are on the same order of magnitude. For kidney slides, a 2D area (1 mm width, Fig. S10) from the cross-section perpendicular to the diameter of the kidney slices was used. The detailed dimensions and computational meshing (i.e., finite element method) can be found in Fig. S10.

We have added Fig. S10 to the Supplementary material:

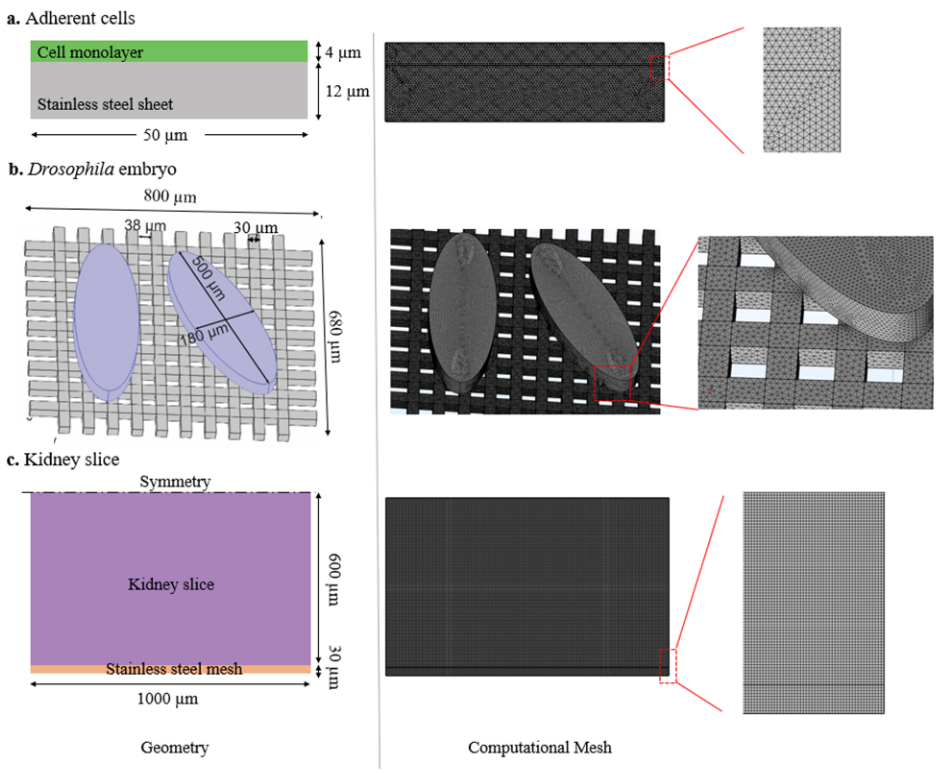

Fig. S10. The geometry and computational mesh used for heat transfer simulation of (a) adherent cells, (b) *Drosophila* embryos and (c) kidney slice in joule heating. The close-up views of the computational meshing are provided.

6. *Moreover, what were the material parameters that were used for the modeling? Parameters like heat capacity and CPA density are temperature dependent, and further change upon phase or state transitions. Have these parameters been derived from extrapolations? Do the material properties of the biosystem (cell /tissue material) also play a role? i.e, properties of the tissue extracellular matrix, and the lipid content in cells. How do these properties affect heat transfer?*
More details are needed to explain how heat transfer simulations were done.

Response: For stainless steel, the constant density (7.8×10^3 kg/m^3) and temperature dependent thermal conductivity and specific heat (obtained from National Institute of Standards and Technology, https://trc.nist.gov/cryogenics/materials/304Stainless/304Stainless_rev.htm) were used in the heat transfer simulation.
For the thermal properties of biosystems (adherent cells, *Drosophila* embryos and kidney slices), please refer to our response to Major questions # 1 from Reviewer 1.

- We have added the following text to page 19 of the manuscript:

For various biosystems (adherent cells, *Drosophila* embryos, kidney slices), in the absence of experimental data on the material properties, we used the thermal properties of relevant samples as previously reported in the

literature. Specifically, temperature dependent density of VS55[50] was used for all biosystems. The reported temperature dependent thermal conductivity of 6 M glycerol-laden porcine liver[42] was used as the estimation for all biosystems. For heat capacity, similar thermal properties have been reported between CPA solutions and CPA-loaded biological samples[21,25,50], we used the experimentally measured values for VS55[25] as the approximation for all biosystems. More detailed characterization of the thermal properties for CPA-laden biosystems (cells, embryos and tissues) is needed in the field, but our simulation should provide an initial approximation of the heat transfer process. For the SS, density was assumed to be constant (7.8×10^3 kg/m^3),

7. *In the MM section, it is mentioned that: 'We used $D = 6.5 \times 10^{-11}$ m^2/s, as reported in a previous study.[21]' What does this number reflect? There are multiple CPAs that were used for vitrification and each component is expected to permeate at a different rate. Moreover, the apparent diffusion coefficient is typically concentration dependent. This should be explained.*

Response: We thank the reviewer for this comment. The effective diffusion coefficient (D, 6.5×10^{-11} m^2/s) was obtained from our experimental measurements of VS55 loading into tissues (i.e., porcine arteries) in a previous study, assuming a constant diffusion coefficient during CPA loading[3]. Therefore, this number represents the overall diffusion rate of each component in VS55 and is assumed to be concentration independent for simplicity. In this study, we use this number to estimate loading of VS55 into kidney slices.

- We have added the following text to page 20 of the manuscript:

D is the effective diffusion coefficient (assumed to be concentration independent for simplicity), and x is the location inside the tissue. We used $D = 6.5 \times 10^{-11}$ m^2/s, as experimentally measured for the loading of VS55 into tissues in a previous study.[21]

8. *The authors compare their findings using joule heating with convective warming results. However, it would be more appropriate to compare the findings here with other warming methods.*

Response: We thank the reviewer for this comment. Our current work focused on the development, characterization and validation of a new platform technology using joule heating to rewarm vitrified and partially vitrified biosystems. Convective warming, as the gold standard method for rewarming in the cryopreservation field, was therefore used for comparison to validate our new joule heating approach. More extensive experimental comparisons between joule heating and other novel warming methods such as RF nanowarming, laser nanowarming, or metal form RF warming etc (Table 1) might distract from the focus of the current work.
In principle, we compared the specific absorption rate (SAR), heating per volume, and reported warming rates of different warming methods in Table 1. Other warming methods fall short in achieving simple, scalable, and rapid rewarming simultaneously in one platform technology for cryopreserving adherent cells, *Drosophila* embryos and tissues.

- We have added the following text to page 13 of the manuscript:

In this study, we validated the performance of joule heating by comparing with convective warming, the gold standard method in the cryobiology field. Future systematic experimental comparisons with other novel warming methods listed in Table 1 will provide more insights into the capacity of the joule heating technique for specific scale systems or applications. In principle, as illustrated in Table 1 comparing the existing novel rewarming methods, joule heating provides the widest range of warming rates in a simple, scalable manner tailored to each biosystems at different scales.

Reference

1. Etheridge, M.L., *et al.* RF heating of magnetic nanoparticles improves the thawing of cryopreserved biomaterials. *Technology* **2**, 229-242 (2014).
2. Joshi, P., Ehrlich, L.E., Gao, Z., Bischof, J.C. & Rabin, Y. Thermal Analyses of Nanowarming-Assisted Recovery of the Heart From Cryopreservation by Vitrification. *Journal of Heat Transfer* **144**, 031202 (2022).
3. Han, Z., *et al.* Diffusion Limited Cryopreservation of Tissue with Radiofrequency Heated Metal Forms. *Advanced healthcare materials* **9**, 2000796 (2020).
4. Choi, J. & Bischof, J.C. Review of biomaterial thermal property measurements in the cryogenic regime and their use for prediction of equilibrium and non-equilibrium freezing applications in cryobiology. *Cryobiology* **60**, 52-70 (2010).
5. Maas, W., *et al.* Assessment of some critical factors in the freezing technique for the cryopreservation of precision-cut rat liver slices. *Cryobiology* **40**, 250-263 (2000).
6. Vanhulle, V., *et al.* Cryopreservation of rat precision-cut liver slices by ultrarapid freezing: influence on phase I and II metabolism and on cell viability upon incubation for 24 hours. *Life Sciences* **68**, 2391-2403 (2001).
7. Fisher, R.L., Hasal, S.J., Lipscomb, J.C., Gandolfi, A.J. & Brendel, K. Cold and cryopreservation of monkey liver slices. *Toxicology Mechanisms and Methods* **12**, 119-133 (2002).
8. de Graaf, I.A., *et al.* Cryopreservation of rat precision-cut liver and kidney slices by rapid freezing and vitrification. *Cryobiology* **54**, 1-12 (2007).
9. Bai, Y., *et al.* Cryopreserved human precision-cut lung slices as a bioassay for live tissue banking. A viability study of bronchodilation with bitter-taste receptor agonists. *American journal of respiratory cell and molecular biology* **54**, 656-663 (2016).
10. Keblinski, P., Cahill, D.G., Bodapati, A., Sullivan, C.R. & Taton, T.A. Limits of localized heating by electromagnetically excited nanoparticles. *Journal of Applied Physics* **100**, 054305 (2006).
11. Qin, Z. & Bischof, J.C. Thermophysical and biological responses of gold nanoparticle laser heating. *Chemical Society Reviews* **41**, 1191-1217 (2012).
12. Elliott, G.D., Wang, S. & Fuller, B.J. Cryoprotectants: A review of the actions and applications of cryoprotective solutes that modulate cell recovery from ultra-low temperatures. *Cryobiology* **76**, 74-91 (2017).
13. Manuchehrabadi, N., *et al.* Improved tissue cryopreservation using inductive heating of magnetic nanoparticles. *Science translational medicine* **9**, eaah4586 (2017).
14. Sharma, A., *et al.* Vitrification and Nanowarming of Kidneys. *Advanced Science* **8**, 2101691 (2021).
15. Zhan, L., Li, M.-g., Hays, T. & Bischof, J. Cryopreservation method for Drosophila melanogaster embryos. *Nature communications* **12**, 1-10 (2021).
16. Rampersad, S.N. Multiple applications of Alamar Blue as an indicator of metabolic function and cellular health in cell viability bioassays. *Sensors* **12**, 12347-12360 (2012).
17. Kang, H.-W., *et al.* A 3D bioprinting system to produce human-scale tissue constructs with structural integrity. *Nature biotechnology* **34**, 312-319 (2016).
18. Loessner, D., *et al.* Functionalization, preparation and use of cell-laden gelatin methacryloyl–based hydrogels as modular tissue culture platforms. *Nature protocols* **11**, 727-746 (2016).
19. Staal, J.A., Alexander, S.R., Liu, Y., Dickson, T.D. & Vickers, J.C. Characterization of cortical neuronal and glial alterations during culture of organotypic whole brain slices from neonatal and mature mice. *PloS one* **6**, e22040 (2011).

REVIEWERS' COMMENTS

Reviewer #1 (Remarks to the Author):

I have confirmed all the points, which I asked to be addressed, are explained well with the acceptable revisions in the main text and Supplemental materials.

Reviewer #2 (Remarks to the Author):

The authors have satisfactory responded to my questions and concerns, and improved their manuscript accordingly.
